# Integrative epigenome profiling of 47XXY provides insights into whole genomic DNA hypermethylation and active chromatin accessibility

**DOI:** 10.3389/fmolb.2023.1128739

**Published:** 2023-03-27

**Authors:** Nan Miao, Zhiwei Zeng, Trevor Lee, Qiwei Guo, Wenwei Zheng, Wenjie Cai, Wanhua Chen, Jing Wang, Tao Sun

**Affiliations:** ^1^ Center for Precision Medicine, School of Medicine and School of Biomedical Sciences, Huaqiao University, Xiamen, Fujian, China; ^2^ Department of Cell and Developmental Biology, Cornell University Weill Medical College, New York, NY, United States; ^3^ United Diagnostic and Research Center for Clinical Genetics, Women and Children’s Hospital, School of Medicine & School of Public Health, Xiamen University, Xiamen, Fujian, China; ^4^ Quanzhou Women and Children’s Hospital, Quanzhou, Fujian, China; ^5^ Department of Radiation Oncology, First Hospital of Quanzhou, Fujian Medical University, Quanzhou, Fujian, China; ^6^ Department of Clinical Laboratory, First Hospital of Quanzhou, Fujian Medical University, Quanzhou, Fujian, China

**Keywords:** klinefelter syndrome, DNA methylation, chromosomal accessibility, ATAC-seq, epigenetics

## Abstract

Klinefelter syndrome (KS, 47XXY) is a disorder characterized by sex chromosomal aneuploidy, which may lead to changes in epigenetic regulations of gene expression. To define epigenetic architectures in 47XXY, we annotated DNA methylation in euploid males (46XY) and females (46XX), and 47XXY individuals using whole genome bisulfite sequencing (WGBS) and integrated chromatin accessbilty, and detected abnormal hypermethylation in 47XXY. Furthermore, we detected altered chromatin accessibility in 47XXY, in particular in chromosome X, using Assay for Transposase-Accessible Chromatin sequencing (ATAC-seq) in cultured amniotic cells. Our results construct the whole genome-wide DNA methylation map in 47XXY, and provide new insights into the early epigenomic dysregulation resulting from an extra chromosome X in 47XXY.

## Introduction

Klinefelter syndrome (KS, 47XXY) is a genetic disorder caused by sex chromosomal aneuploidy in individuals carrying a 47, XXY karyotype (Groth et al., 2013; Samango-Sprouse et al., 2016; Los and Ford, 2022). The supernumerary chromosome X (Chr.X) affects testicular development and results in infertility and hyper gonadotropic hypogonadism in adults (Davis et al., 2015; Davis et al., 2019). Generally, 47XXY individuals have low levels of serum follicular stimulating hormone (FSH) and luteinizing hormone (LH), and a diffused hyalinization of seminiferous tubules as well as absence of mature Leydig cells (Bonomi et al., 2017). The hyper gonadotropic hypogonadism is tightly linked to some features such as increased prevalence of diabetes, obesity and metabolic syndrome, osteoporosis and cognitive disabilities in 47XXY (Grace, 2004; Lanfranco et al., 2004).

Studies have shown that phenotypic features seen in 47XXY are likely associated with overexpression of some Chr.X genes escaping X chromosomal inactivation due to a dosage effect (Carrel and Willard, 1999; Carrel and Willard, 2005; Balaton et al., 2015; Belling et al., 2017; Navarro-Cobos et al., 2020). Increased copy number of *SHOX5*, which is localized in the pseudoautosomal region (PAR) in Chr.X, is likely a cause of the tall stature in 47XXY individuals (Ottesen et al., 2010; Ibarra-Ramirez et al., 2020). Moreover, the supernumerary Chr.X may cause widespread changes in the methylome in 47XXY children and adults (Viana et al., 2014; Sharma et al., 2015; Wan et al., 2015; Skakkebaek et al., 2018; Zhang et al., 2020). CpG sites annotated to HEN1 methyltransferase homolog 1 (*HENMT1*), calcyclin-binding protein (*CACYBP*), and GTPase-activating protein (*SH3* domain)-binding protein 1 (*G3BP1*) genes have been reported within 47XXY-specific loci, suggesting an epigenetic regulation in 47XXY (Wan et al., 2015). Gene expression differences and transcriptomics sequencing have been extensively studied (Astro et al., 2021; Davies, 2021; He et al., 2022). The azoospermia factor (AZF) microdeletions is considered the most frequent genetic cause of male infertility along with Klinefelter syndrome (Lahoz Alonso et al., 2023). Based on RNA-seq and scRNA-Seq data, KIF2C may be closely related to the differentiation and development of sperm cells in 47XXY (He et al., 2022). However, there is still much unknown about how epigenetic regulatory landscape evolve in 47XXY.

To explore the epigenetic pathogenesis of chromosome aneuploidy, several lines of research have focused on DNA methylation pattern changes (Laufer et al., 2019; Laufer et al., 2021). Based on the single base methylation patterns, bisulfite sequencing has been widely used, thus allowing genome-wide measurement of DNA methylation levels (Beck and Rakyan, 2008; Li et al., 2018). Epigenetic modifications of chromatin determine TF-binding site accessibility at gene promoters and regulatory elements (Pombo and Dillon, 2015; McLaughlin et al., 2019). Chromatin accessibility mapping is a powerful approach to identify potential regulatory elements. A popular example is Assay for Transposase-Accessible Chromatin using sequencing (ATAC-seq), whereby Tn5 transposase inserts sequencing adapters into accessible DNA (“tagmentation”) (Buenrostro et al., 2015; Shashikant and Ettensohn, 2019; Henikoff et al., 2020). ATAC-seq can help understand the precise chromosomal open ability, for instance in cells from 47XXY. All these studies have proven that comparative epigenomics is a powerful tool to investigate the molecular basis of 47XYY. To complement this resource, we have processed whole-genome bisulfite sequencing data, as well as chromatin accessibility data.

In this study, to obtain a comprehensive single-base DNA methylation landscape of 47XXY, we conducted WGBS from the plasma samples of 47XXY, 46XX and 46XY. We found that the 47XXY shows whole genome abnormal hypermethylation changes, compared to 46XY. To explore the chromatin accessibility at fetal stages, we performed ATAC-seq using amniotic cells. We found that the 47XXY shows a dynamic chromatin accessibility than 46XY and 46XX. We observed the correlation between chromatin contact and methylation maps from fetal stages to adulthood.

## Results

### Extra chromosome X causes changes of the whole genome DNA methylation level in 47XXY

**FIGURE 1:**
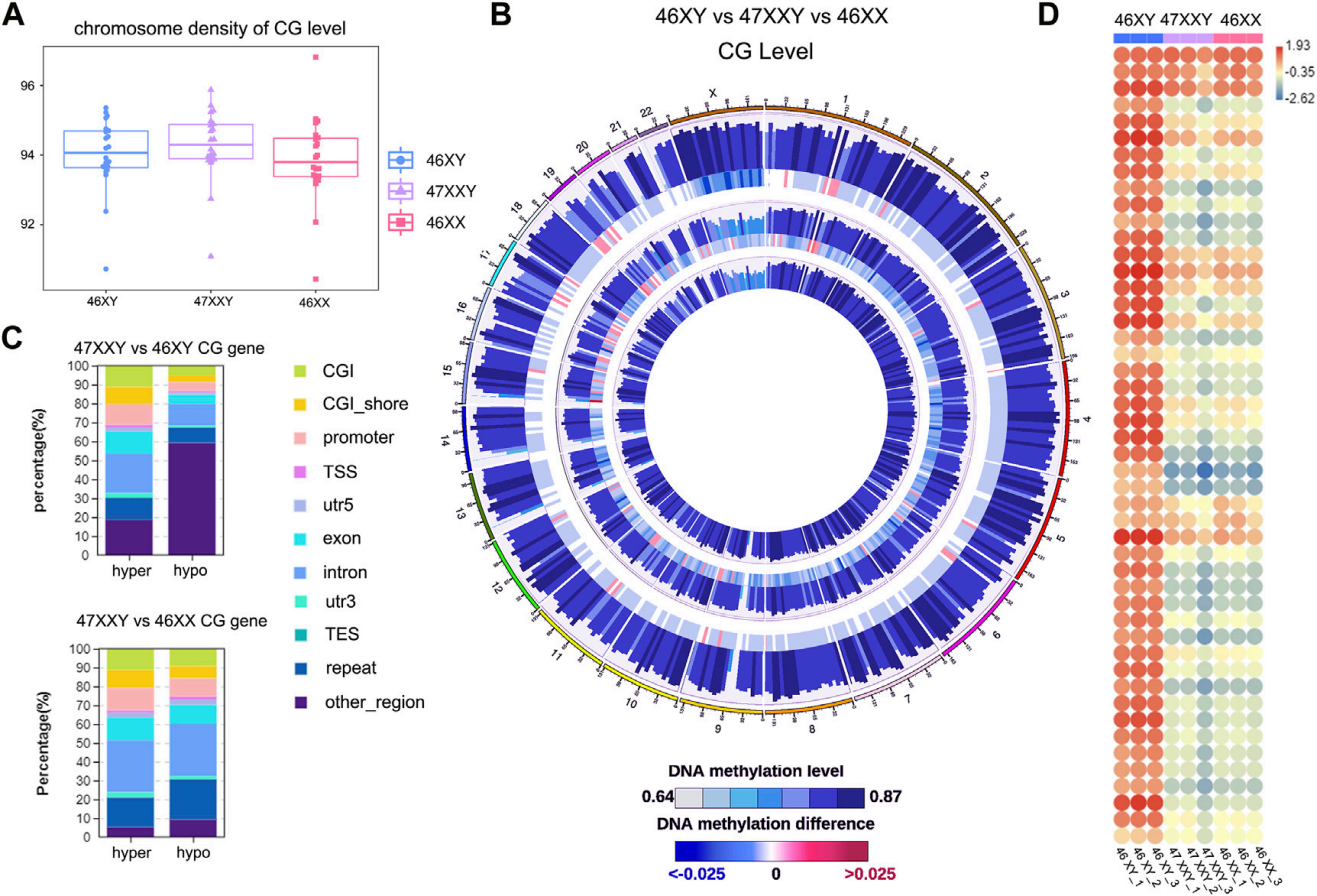
The single base methylation level of 47XXY, 46XY and 46XX in CG, CHG and CHH. **(A)** The beeswarm of chromosome density analyses on of the CG level within the genome-wide scope of 46XY, 47XXY and 46XX. The horizontal axis represents different sample/group names, and the vertical axis represents the methylation level, with 10 KB as a bin. **(B)** Circos diagram of comparing the methylation levels of different groups. From the outside to the inside, the five circles indicate the methylation level in the 46 XY group, the methylation level difference between the 46XY and 47XXY groups, the methylation level in the 47XXY group, the methylation level difference between the 47XXY and 46XX groups and the methylation level in the 46XX group. The methylation levels in 46XY, 47XXY and 46XX groups, Internal scale: DNA methylation level indicates the level of methylation, while DNA methylation difference indicates the level of methylation difference between samples. **(C)** The Stacked Column of hyper and hypo CG genes in functional regions: CGI, CGI_shore, promoter, 5’ untranslated region (5’UTR), exon, intron, 3’ untranslated region (3’UTR) and repeats region. **(D)** The heatmap of methylation level in Chr. X in 46XY, 47XXY and 46XX. Each circle represents the everage methylation level in 48 regions of Chr.X. The colors from red, white to blue mean the methylation levels from high, median to low (1.93, −0.35 to −2.62).

To better understand the impact of an extra Chr.X on methylation regulation, we constructed a genome wide methylome map using WGBS, from plasma samples in 3 euploid males (46XY), 3 euploid females (46XX) and 3 47XXY individuals (Figure 1).

Firtly, We assessed sequencing quality, annotated sequence alignment of WGBS results, and analyzed methylation sites using Bismark (Supplementary Tables S1–S4) (Krueger and Andrews, 2011). Studies have shown that DNA methylation is predominantly detected at CpG sites (CG), and is much less commonly observed at non-CpG sites (CHH and CHG, H represents A, C, T) (Holliday and Pugh, 1975; Coulondre et al., 1978; Bird, 1980; Jang et al., 2017). We first examined the CG level with hyper or hypo methylation methylation within the genome-wide scope of each group by performing chromosome density analyses. The 47XXY group showed highest CG level than those in 46XY and 46XX groups (Figure 1A). To visualize 9 samples’ grouping and correlations, the principal components analysis (PCA) and Pearson analysis were performed (Supplementray Figure S1). Both PCA and Pearson analyses indicate that the datasets of 47XXY show a better correlation with that of 46XX than that of 47XY (Supplementray Figure S1).

We next compared the overall methylation levels among the three groups (Figure 1B). To explore more details of CG levels with the chromosomal density, we counted 902 chromosomal regions by Circos diagram, and found 69 regions showing significant differences in 47XXY vs. 46XY, 128 regions with significant differences in 47XXY vs. 46XX (Supplementray Table S5 and Figure 1B). Interestingly, the CG levels displayed hyper/hypo methylation in Chr. 1, Chr. 2, Chr.6, Chr.9-12, Chr15-22, in particular in Chr.X (Figure 1B). The CG levels in samples of 47XXY were different from those in the other two groups, particularly on Chr.1 and Chr.X (Supplementray Figure S2). We calculated the average CG level in chromosomes, and found that 47XXY shows significantly higher level in Chr.X than that in 46XY, and the average CG level shows no significant differences in whole genome in 47XXY vs. 46XX (Supplementray Table S5). Moreover, all samples showed high CG levels in the intron, 3’-untranslated region (3’-UTR) and repeats region, medial CG levels in the CpG island shores (CGI shores), promoter and exons, and low methylation levels in the CpG island (CGI) and 5’-UTR (Supplementray Figure S2 **)**. These results suggest that the 47XXY group shows whole genome-wide suppression, compared with 46XY and 46XX groups.

The CG genes with differential methylation levels have been detected in 47XYY that compared to 46XY males and 46XX females (Figure 1C). In 47XXY vs. 46XY, there were more hyper CG genes in the promoter, exon and intron, and more hypo CG genes (59.11%) in other un-defined genomic regions (Figure 1C). Compared to 46XY males, large proportions of CG genes in 47XXY were located in unknown regions in chromosomes. In 47XXY vs. 46XX, there were more hyper CG genes than hypo CG genes in the promoter, exon and 3’UTR regions (Figure 1C).

Next, the CG levels of Chr. X were constructed, separately **(** Figure 1D). The X-linked methylation level in 47XXY is lower than the 46XY, and much closer to 46XX (Figure 1D). In addition, the 47XXY individuals showed a similar methylation landscape to that of the 46XX, and fewer methylation sites than 46XY (Figures 1C, D).

Based on the CG methylation analysis in three groups, our analyses demonstrate that the 47XXY adult blood methylome is distinguishable from that of both 46XY and 46XX. In two comparisons, we found more CG genes in 47XXY vs. 46XY. Compared to 46XX and 46XY, 47XXY individuals show a similar methylation landscape to the 46XX females.

### The DMR level in 47XXY have significanly changed

**FIGURE 2:**
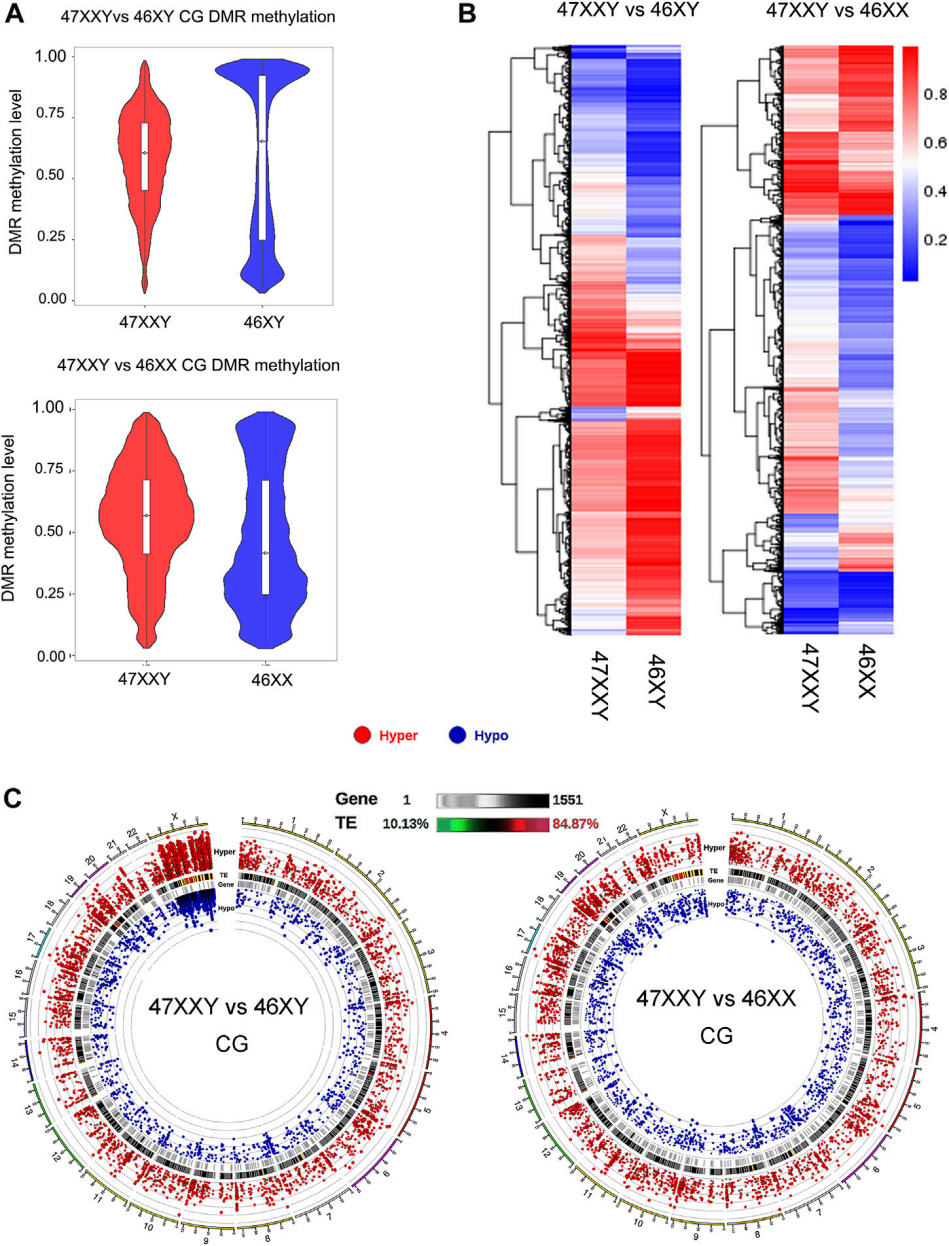
Comparison analysis in CG DMR genes in two groups (47XXY vs. 46XY and 47XXY vs. 46XX) **(A)** The violin map of horizontal distribution in CG DMR methylation (47XXY vs. 46XY and 47XXY vs. 46XX). The X-coordinate represents the comparison group, and the Y-coordinate represents the methylation level value. The distribution of DMR methylation level is shown in the form of violin plot (boxplot inside and the quantity distribution under this methylation level on the flank). **(B)** The heatmap of clustered CG DMR methylation level. The horizontal axis represents the comparison group, the vertical axis represents the methylation level value clustering effect, and the blue to red represents the methylation level from low to high. **(C)** Circos map of CG DMR methylation genes in two comparison (47XXY vs. 46XY and 47XXY vs. 46XX). From outside to inside: (1) Hyper DMR statistic log_5_^(|areaStat|)^; The higher the outward dot is, the more significant the position difference is. The red circle represents hyper CG DMR. (2) TE, and the heat map of the proportion of repeat original. (3) Gene density heat map. (4) Hypo DMR statistic log_5_^(|areaStat|)^.

Genomic regions showing different levels of DNA methylation under distinct biological conditions are termed as “differentially methylated regions, DMRs”, which have been found in the embryonic reprogramming and developmental phases (Reik et al., 2001; Meissner et al., 2008; Doi et al., 2009). The CG methylation is known to dominate mammalian genomes. However, the non-CG methylation (CHG and CHH, where H refers to A, C, or T) is commonly observed in plants and fungi (Lu et al., 2023). To explore the difference in 47XXY vs. 46XY and 47XXY vs. 46XX, we calculated the number of DMR with a mean beta value difference of at least 0.25 (Supplementray Figure S3). Most of the DMR in CHG and CHH was excluded. We further analyzed the methylation length distribution, average methylation level, and DMR status by using dispersion shrinkage for sequencing data (DSS) (Figure 2, Supplementray Figures S4–S8) (Feng et al., 2014; Wu et al., 2015; Park and Wu, 2016).

The average DMR methylation level in the CG in the 47XXY group was lower (about 0.5) than that in the 46XY group, and higher than that in the 46XX group (about 0.35) (Figure 2A and Supplementray Figure S5). In a comparison of 46 XY vs. 46 XX, the average methylation level of 46XY (over 0.75) was higher than that in 46XX (over 0.5) (Supplementray Figure S5). Above all, the DMR CG level of 47XXY is in between those in 46XY and 46XX.

We also compared different DMRs in CG, CHG and CHH regions of annotated genes among three groups (Figure 2B and Supplementray Figure S6). In the 47XXY, most of the annotated genes (CG, CHG and CHH) show a lower average methylation level than those in the 46XY group, in which these genes displayed higher average methylation levels than those in the 46XX groups (Figure 2B and Supplementray Figures S7, S8). These results reported an unbalanced methylation in 47XXY, in particular in the CG site.

Compared to 46XY, the hyper and hypo CG DMRs of 47XXY were abundant in Chr.1 (804 & 241), Chr.19 (697 &198) and Chr.X (598 & 657) (Figure 2C and Supplementray Table S6). Moreover, a large proportion of these CG DMRs was located in the genetic repeat region of Chr.X. In 47XXY vs. 46XX, Chr.1 (777 & 264), Chr.19 (816 & 246), and Chr.17 (646 & 192) were the top 3 highly altered chromosomes (Figure 2C and Supplementray Table S7). In Chr.1, Chr.16 and Chr.19, the hyper/hypo CG DMRs were located in gene-enriched regions. However, only a few Y linked CG DMRs were changed in 47XXY, as compared to 46XY. Noticeably, a large number of CG DMRs in 47XXY was located in the Chr.X. While it is not possible to directly infer gene expression changes from methylation data, we categorized the DMRs as likely inhibitory, possible inhibitory, or unknown concerning expected effect on gene expression.

Thus, the CG DMRs analysis indicates that the methylation levels in 47XXY have significantly changed, especially in Chr.X.

### Chromatin accessibility displays changes in amniocytes of 47XXY fetuses

Based on WGBS results, we found that the extra Chr. X causes global DNA methylation changes in the 47XXY. The epigenome, including DNA methylation, is substantially reprogramed during the gestational period, resulting in epigenetic modifications that are stable and maintained throughout life. ATAC-seq identifies nucleosome-depleted (open) chromatin, which harbors potentially active gene regulatory sequences, we thus analyzed the chromatin accessibility in the fetal amniocyte by ATAC-seq.

**FIGURE 3:**
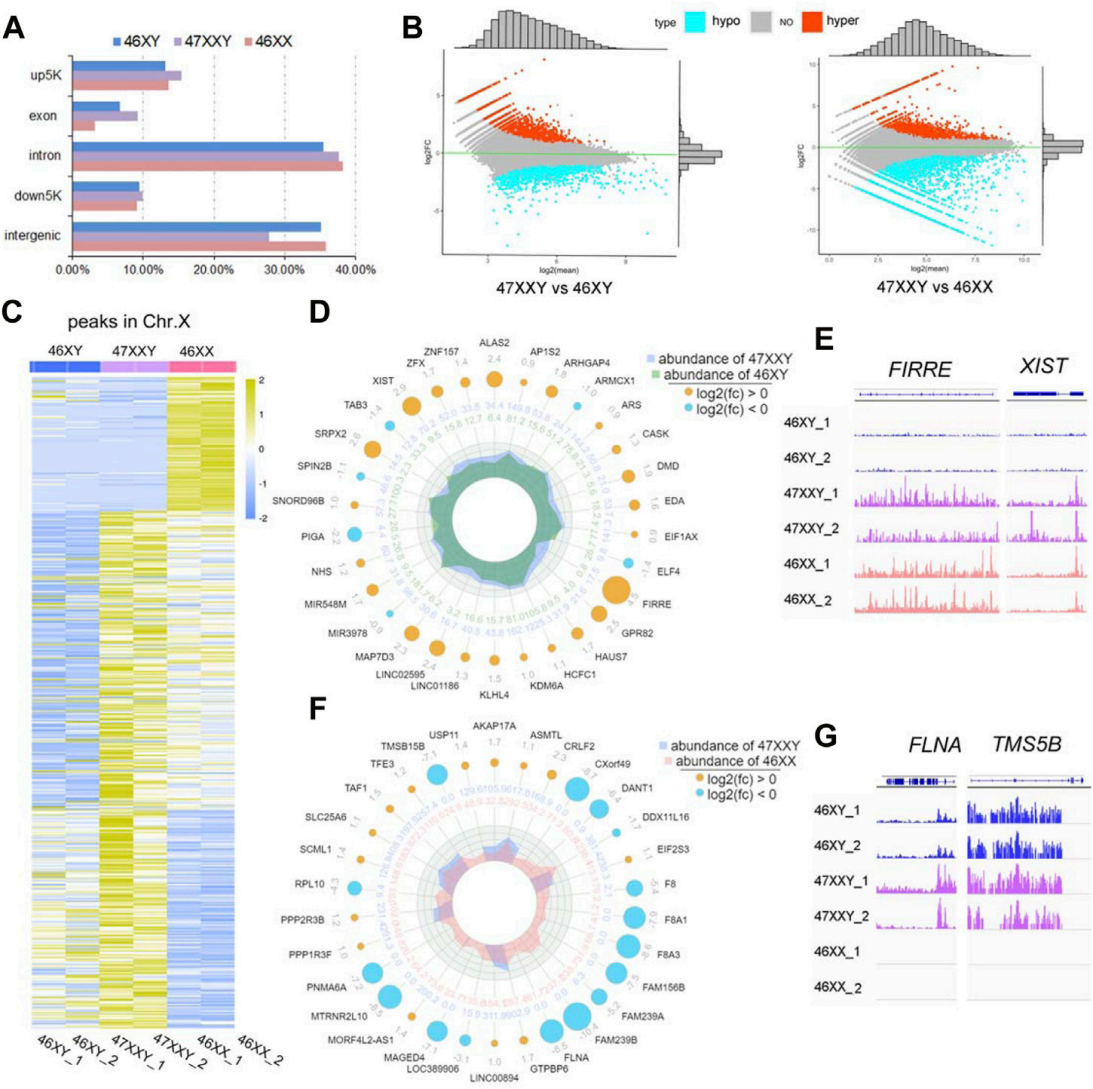
ATAC-seq of 6 amniotic samples (46XY, 47XXY and 46XX). **(A)** The bar plot of the proportion in different functional elements in 46XY, 47XXY and 46XX (up5K, exon, intron, down5K, and intergenic). **(B)** MAplot map of two combinations (47XXY vs. 46XY, 47XXY vs. 46XX). The *X*-axis represents log_2_ mean normalized-counts and *Y*-axis represents log_2_^fold-change^. **(C)** The heatmap of the peaks in Chr. X of 46XY, 47XXY and 46XY groups. The blue to yellow represent the methylation level from low to high. **(D)** Radar circus of the most significant hyper and hypo genes of Chr. X in 47XXY vs. 46XY. From outside to inside: gene name with log_2_^(fold-change)^. **(E)** IGV illustration map of hyper open abilities in Chr.X (46XY vs. 47XXY vs. 46XY). **(F)** Radar circus of the most significant hyper and hypo genes of Chr.X in 47XXY vs. 46XX. From outside to inside: gene name with log_2_^(fold-change)^. **(G)** IGV illustration map of hypo open abilities in Chr.X (46XY vs. 47XXY vs. 46XY). *FLNA* and *TMSB15B* were the specific peak genes in 46XY and 47XXY, and the empty signals mean that they are found in 46XX.

Firstly, ATAC-seq data were analyzed by performing pre-alignment QC, read alignment to a reference genome, and post-alignment QC and processing (Yan et al., 2020). The annotated “peaks” reflect different levels of chromatin accessibility in gene expression regulation (Tarbell and Liu, 2019; Yan et al., 2020). Thus, we obtained high quality reads (the clean rates are over 97%) and analyzed peaks (Supplementray Tables S8–S10). To visualize grouping and correlations of 6 samples, the principal components analysis (PCA) and Pearson analysis were performed (Supplementray Figure S9). Both PCA and Pearson analysis indicate that the dataset of 47XXY shows a better correlation with that of 46XX than that of 47XY (Supplementray Figure S9 and Supplementray Table S9). Compared to both 46XY and 46XX, the peaks in 47XXY subjects showed the highest proportion in up5K (46XY: 13.11%, 47XXY: 15.44% and 46XX: 13.62%), exon (46XY: 6.77%, 47XXY: 9.20% and 46XX: 3.21%), and down5K (46XY: 9.48%, 47XXY: 9.93% and 46XX: 9.13%), and the lowest proportion in intergenic (46XY: 35.81%, 47XXY: 27.80% and 46XX: 35.14%) (Figure 3A, Supplementray Figure S10 and Supplementray Table S10). These results suggest that the 47XXY shows more active chromatin accessibility than both those in 46XX and 46XY.

In 47XXY vs. 46XY, 3790 peaks showed upregulated (high-accessible) (log_2_^fold-change (47XXY vs 46XY)^ > 0) and 5600 peaks displayed hypo (log_2_^fold-change (47XXY vs 46XX)^ < 0) (Figure 3B and Supplementray Table S11). Compared to 46XX, 5824 peaks displayed upregulated and 4261 peaks showed hypo regulated (Figure 3B and Supplementray Table S12).

In addition, a large proportion of X chromosomal peaks in 47XXY was higher than that in 46XX and 46XY (Figure 3C). In comparisons of 47XXY vs. 46XY and 47XXY vs. 46XX, maximal differences of X linked peaks in annotated genes were shown in a circular radar map (Figures 3D, E, Supplementray Tables S11, S12). There were 26 upregulated annotated peak genes (such as *XIST, ZFX* and *FIRRE*), 4 hypo X-linked annotated peak genes (*TAB3*, *SPIN2B*, *PIGA* and *miR39*) were detected in 47XXY vs. 46XY (Figure 3D). In 47XXY vs. 46XX, 14 upregulated X-linked annotated peak genes (such as *USP11, AKAP17A, ASMTL* and *CRLF2*) and 16 hypo (such as *TMSB15B*, *DANT*, *FLNA* and *FAM239B*) were detected (Figure 3F). Among two comparisons, *FIRRE* and *XIST* in 47XXY retained an open chromatin than those in 46XY (Figure 3E); and peaks in *FLNA* and *TMSB15B* were higher in 47XXY than those in 46XX (Figure 3G).

ATAC-seq analysis revealed that the chromatin in the vicinity of the peak genes in 47XXY are abnormally active, especially in Chr. X, more active than those in 46XY and 46XX.

### Correlation between the methylation level and chromatin accessibility in 47XXY

**FIGURE 4:**
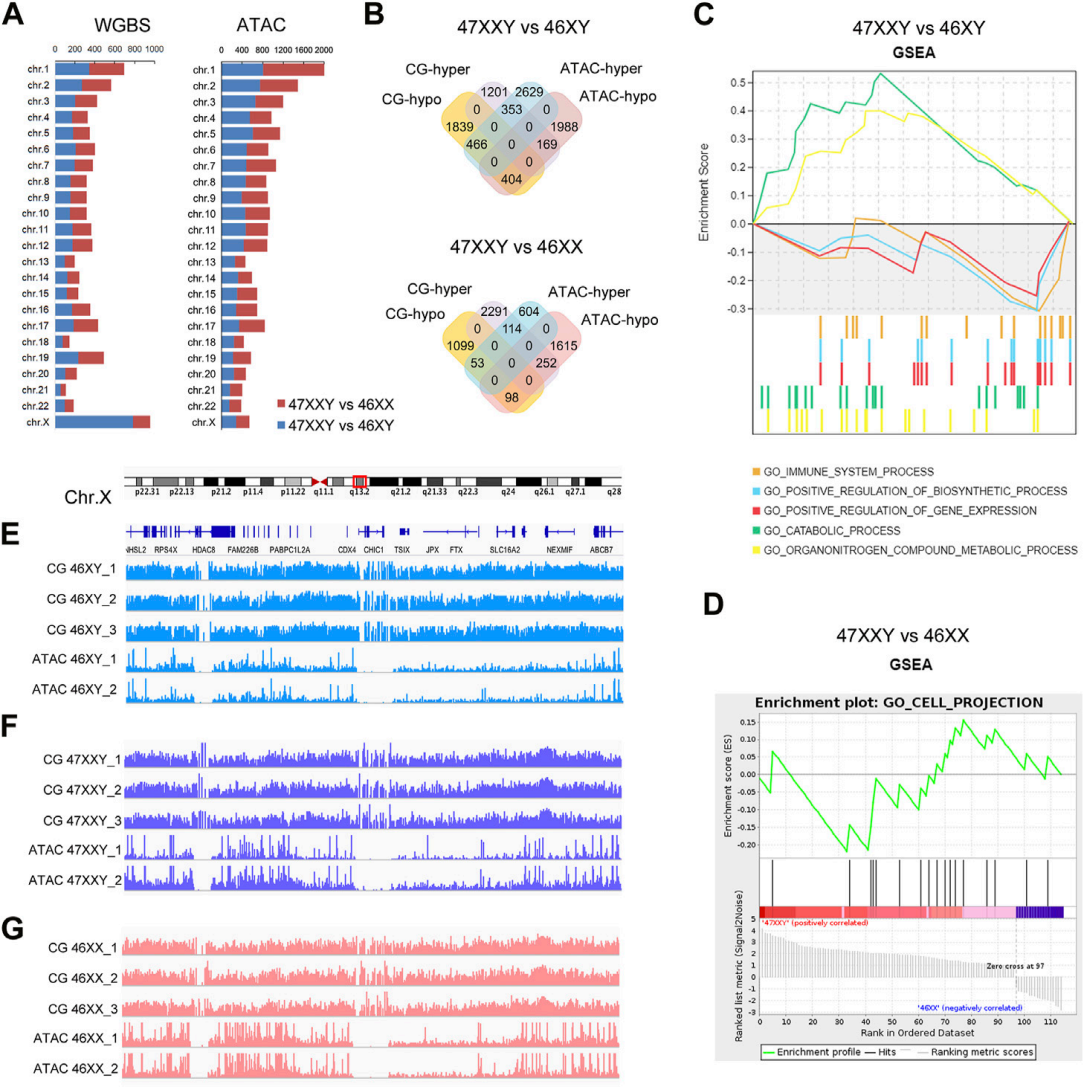
The combination result of WGBS and ATAC-seq **(A)** Chromosome location number in 47XXY vs. 46XY, 47XXY vs. 46XX of WGBS and ATAC-seq **(B)** Venn diagrams of hyper and hypo CG DMR anchoring genes and ATAC-seq in 47XXY vs. 46XY, 47XXY vs. 46XX. **(C)** GSEA analysis in combination data of two series (WGBS and ATAC-seq) in 47XXY vs. 46XY. **(D)** GSEA analysis in combination data of two series (WGBS and ATAC-seq) in 47XXY vs. 46XX. **(E–G)** IGV map of Chr.X q13.2 in two series data (WGBS and ATAC-seq) from 46XY, 47XXY and 46XX groups.

To explore the integrative epigenetic mechanism of 47XXY, we compared and combined the two data sets of WGBS and ATAC-seq (Figure 4, Supplementray Tables S11–S16).

We first mapped various differentially methylated genes or differentially accessible genes in different chromosomes of two comparisons (47XXY vs. 46XY, 47XXY vs. 46XX) (Figure 4A). From WGBS result, we found: the number of autosomal DMR CG genes were similar; in Chr.X, there are 784 DMR CG genes in 47XXY vs. 46XY, and 174 CG genes 47XXY vs. 46XX (Figure 4A). From the ATAC-seq, the numbers of peak genes in Chr.2-6, Chr.8, Chr.10, Chr.11, Chr.13 and Chr. X in the comparison of 47XXY vs. 46XY were more than those in that of 47XXY vs. 46XX (Figure 4A).

Because a high methylation level corresponds to downregulated chromatin accessibility (Lhoumaud et al., 2019; Spektor et al., 2019), we analyzed genes hyper CG and hypo peaks (termed as hyper gene) and *vice versa* (termed as hypo gene). In 47XXY vs. 46XY, there were 169 hyper genes, and 466 hypo genes (Figure 4B and Supplementray Tables S11–S16). Between 47XXY and 46XX, 252 hyper genes and 53 hypo genes were found (Figure 4B and Supplementray Table S16). These results suggest an association of early imbalanced chromatin activity and the adult methylation level in 47XXY.

We next explored potential functions of these genes using Gene Set Enrichment Analysis (GSEA) (Figures 4C, D). In 47XXY vs. 46XY, the hyper genes were enriched in catabolic process and organ nitrogen compound metabolic process, and hypo genes were enriched in immune system, positive regulation of biosynthetic process and positive regulation of gene expression (Figure 4C). In 47XXY vs. 46XX, only the hypo genes were enriched in cell projection (Figure 4D).

We next visualized DNA methylation and chromatin accessibility at distal regions of genes in q13.2 of Chr.X (Figures 4E–G). We observed greater chromatin accessibility at distal regions corresponding to the lower DNA methylation (Figures 4E–G). These results suggest a correlation between the methylation level and the chromatin accessibility, in particular in Chr.X.

### Altered methylation level and chromatin accessibility in Chr.Y

Based on known RNA-seq data of 47XXY, we next explored the DNA methylation and chromatin accessibility of XXY individuals in four types of sex-chromosome genes: (i) X-linked genes that undergo Chr. X inactivation (XCI), (ii) X-linked genes that escape XCI (XCIE), (iii) pseudoautosomal region (PAR) genes, and (iv) Y-linked genes (Deng et al., 2014; Raznahan et al., 2018).

**FIGURE 5:**
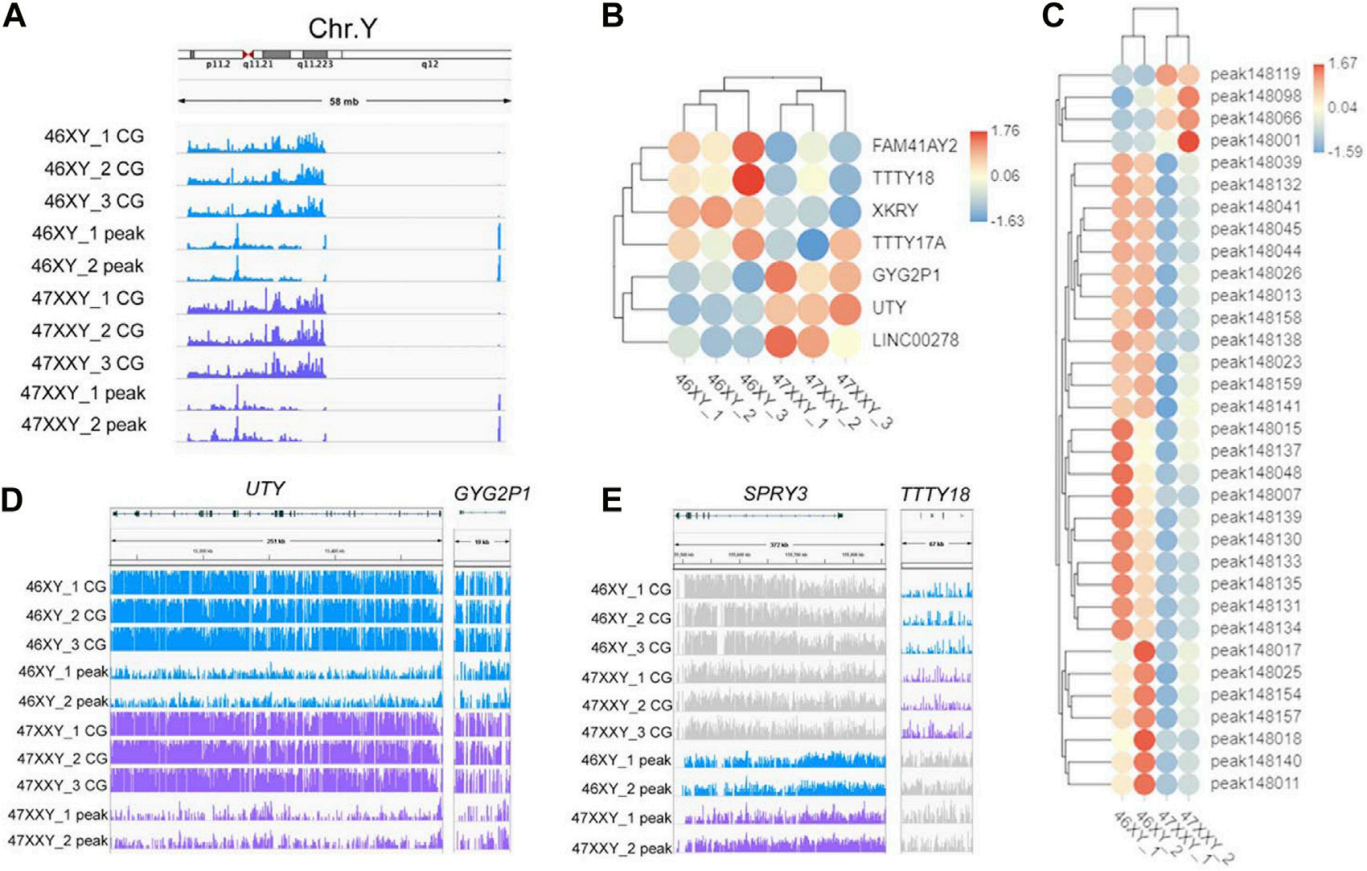
The methylation level and chromatin accessibility of the Chr.Y **(A)** IGV map of Chr.Y (58 Mb) in two series data (WGBS and ATAC-seq) from 46XY and 47XXY groups. The blue channel represents the 46XY, and the purple channel represents the 47XXY, and the 46XX has a little signal. **(B)** Heatmap of the methylation level in Chr.Y. Each circle represents the normalized methylation level in each sample in 46XY and 47XXY. The colors from red, white to blue mean the methylation levels from 1.76, 0.06 to −1.63. **(C)** Heatmap of the chromatin accessibility in Chr.Y. Each circle represents the normalized chromatin open level in each sample in 46XY and 47XXY. The colors from red, white to blue mean the methylation levels from from high, median to low (1.67, 0.03 to −1.59). **(D)** IGV map of *UTY* and *CYG2P1* gene. **(E)** IGV map of *SPRY3* and *TTTY18* genes.

To explore whether the extra Chr.X can influence the Y chromosome, we analyzed the Y-linked genes between the 47XXY and 46XY individuals (Figure 5). Expression levels of Y-linked genes such as *CYorf15B, DDX3Y, NLGN4Y, TMSB4Y, USP9Y, UTY* and *ZFY* showed no significant differences in methylation and chromatin accessible analyses between 46XY and 47XXY (Figure 5A). Eight CG genes (*ARHGEF7, ATP8B4, Fam236A, GAD2, LMTK3, MIR205HG, LALBA* and *MAGED1*) and seven peak genes (*USP9Y, SPRY3, NLGN4Y, TTTY15, TTTY16, GYG2P1* and *BCORP1*) displayed significant changes in the DMR region and in chromatin accessible level, respectively (Figures 5B, C). The *UTY* and *CYG2P1* gene showed changes in the CG intron site, and the *SPRY3* and *TTTY18* displayed no obvious changes (Figures 5D, E and Supplementray Table S16). These results suggest that the extra X chromosome does not significantly alter the Y chromosomal epigenome activity in 47XXY.

### Methylation level and chromatin accessibility in Chr.X

**FIGURE 6:**
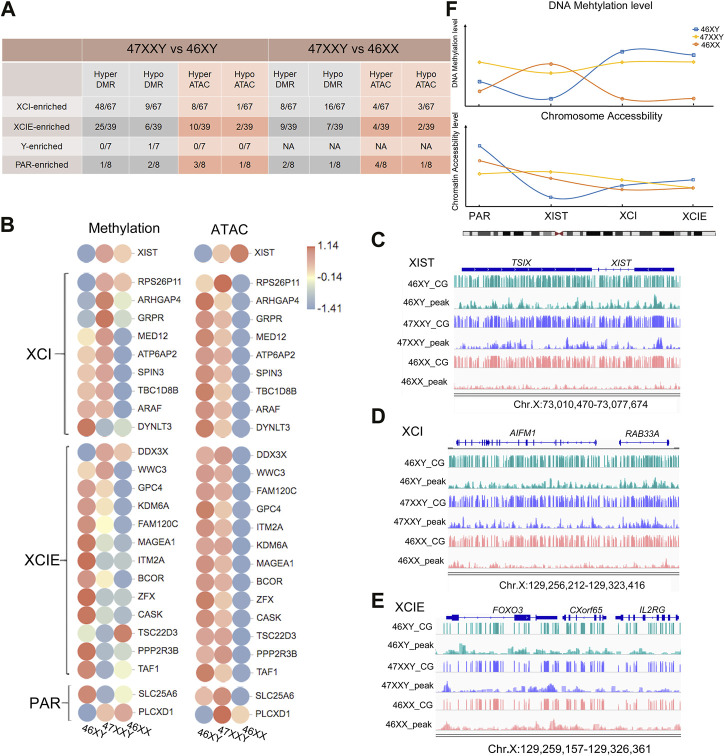
The overlapped genes in XIST, XCI, XCIE and PAR region. **(A)** Statistical data in different functional regions of the Chr.X (XIST, XCI, XCIE and PAR). **(B)** The heatmap of XIST, XCI, XCIE and PAR genes in WGBS and ATAC-seq. Each circle represents the normalized methylation level/open ability in each sample in 46XY, 46XX and 47XXY. The colors from red, white to blue mean the methylation levels from high, median to low (1.14, −0.14 to −1.41). **(C–E)** IGV map of XIST, XCI and XCIE genes in WGBS and ATAC-seq. The blue channel represents 46XY, and the purple channel represents 47XXY, and the pink chanel represents the 46XX. **(F)** The mean methylation level of XIST, XCI, XCIE and PAR genes. The blue hollow square represents 46XY, the yellow hollow rhombus represents 47XXY and the orange hollow circle represents 46XX. The corresponding lines represent the methy trend in XIST, XCI, XCIE and PAR genes.

First, we counted the hyper and hypo genes in 47XXY vs. 46XY and 47XXY vs. 46XX. Compared to the 46XY, the 47XXY group showed a large proportion of upregulated genes (48/67) in the XCI region based on WGBS, and 8 upregulated genes based on the ATAC result. A large proportion of CG genes (25/39) was upregulated in the comparison of 47XXY vs. 46XY in the XCIE region based on the WGBS result, and 10 hyper and 2 hypo peak genes based on the ATAC result. Moreover, 62.5% peak genes were hyper or hypo in the PAR genes based on the ATAC result (Figure 6A and Supplementray Table S16). These results indicate that genes in the XCI and XCIE regions of Chr.X are significantly altered in 47XXY individual.

We next constructed a heatmap of the high CG genes with hypo peaks in different Chr. X regions (Figure 6B). Interestingly, the *XIST* gene displayed a relatively low level of methylation and chromatin accessibility in 46XY samples, and a high level of chromatin accessibility in 47XXY individuals (Figure 6B). Most of the genes in the XCI-enriched region such as *MED12, ATP6AP2, SPIN3, TBC1D8B* and *ARAF* in the 47XXY group showed a higher expression score than those in the 46XX group (Figure 6B). Furthermore, the *XIST* and *ARHGAP4* genes in 47XXY showed the highest methylation level, compared to those in 46XY and 46XX based on the IGV illustration map (Figures 6C, D). And *DDX3X* displayed the highest methylation level and ATAC level in 47XXY (Figure 6E). These results indicate that the XCI genes of 47XXY individuals have similar methylation and ATAC levels to those of the 46XY, and much higher levels than those of the 46XX.

We next analyzed the average methylation and chromatin accessible in the PAR, XIST, XCI and XCIE regions. We found that the methylation level of XXY individuals was in between those of 46XX and 46XY. The chromatin accessible levels of *XIST* and XCIE genes in 47XXY were similar to that in 46XX (Figure 6F).

In summary, these results show that the *XIST* gene in 47XXY has a similar trend to that in 46XX; and the XCI and XCIE genes in 47XXY are similar to those in 46XY, and the XCI genes are higher than those in 46XX.

### Abnormal hormone pathways in 47XXY

**FIGURE 7:**
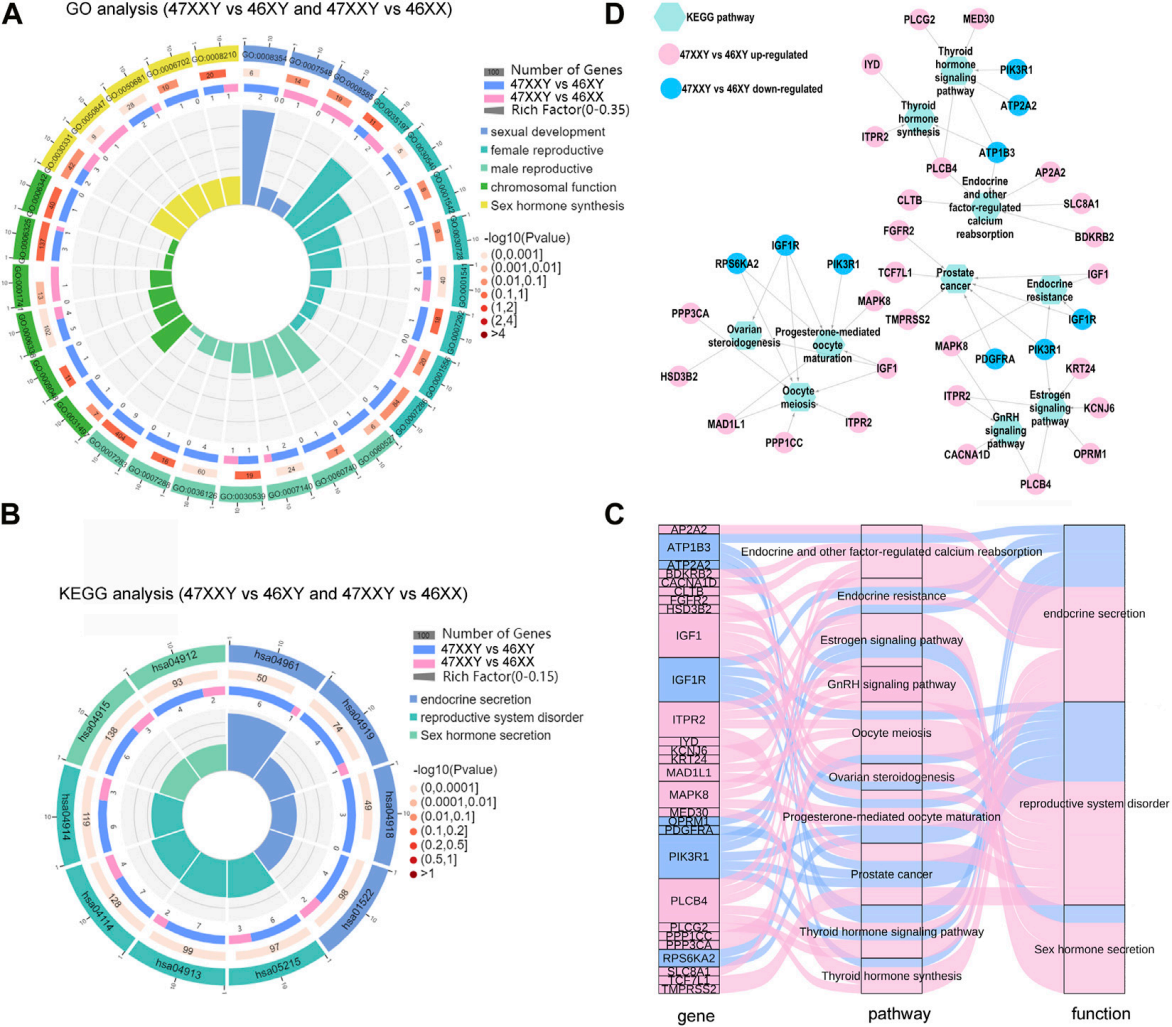
The hormone secretion pathway of the overlapped genes in WGBS and ATAC-seq. **(A)** Comparison analysis of GO items of sexual related biological process in 47XXY vs. 46XY and 47XXY vs. 46XX. **(B)** Comparison analysis of hormone secretion related KEGG pathways in 47XXY vs. 46XY and 47XXY vs. 46XX. Classified as 3 functions: 1. Endocrine secretion (Endocrine and other factor-regulated calcium reabsorption, Endocrine resistance, Thyroid hormone signaling pathway and Thyroid hormone synthesis); 2. Sex hormone secretion (Estrogen signaling pathway, GnRH signaling pathway); 3. reproductive system disorder (Oocyte meiosis, Ovarian steroidogenesis, Prostate cancer, Progesterone-mediated oocyte maturation) **(C)** Sanky map of overlapped genes in different pathways. **(D)** Network of the genes (47XXY vs. 46XY and 47XXY vs. 46XX) in different hormone secretion pathways.

Studies have shown that hormonal imbalance with hyper gonadotropic hypogonadism is a major reason of gonadal dysfunction in 47XXY individuals (Grace, 2004; Lanfranco et al., 2004). We next performed functional analyses to reveal CG and peak genes associated with different hormonal secretions in 47XXY by using Gene Ontology (GO) and Kyoto Encyclopedia of Genes and Genomes (KEGG) analyses (Figure 7) (Xie et al., 2011). The combined genes were performed in the Kobas 3.0 (http://kobas.cbi.pku.edu.cn/).

In the GO analysis, genes with high DNA methylation level and low chromatin accessibility were involved in developmental process, nervous system development, and cell development and biogenesis (Supplementray Tables S17, S18 and Supplementray Figure S11). In comparisons of 47XXY vs. 46XY and 47XXY vs. 46XX, we selected 33 GO items of five different functions: sexual development, female/male reproductive system development, chromosomal function and sexual hormone secretion (Figure 7A and Supplementray Figures S19, S20). 15 items such as germ cell migration, spermatid development, sperm flagellum, and spermatogenesis were enriched in the comparison between 47XXY and 46XY. 5 items such as sex differentiation, XY body, and female gonad development were enriched in the comparison of 47XXY vs. 46XX. Compared to 46XY and 46XX, we found that the overlapped gene in 47XXY males were enriched in sexual related biological process (Figure 7B and Supplementray Tables S19, 20).

From the pathway enrichment analysis (KEGG) of overlapped gene in WGBS and ATAC, we selected 10 hormone secretion and reproductive related pathways in two comparisons (47XXY vs. 46XY and 47XXY vs. 46XX), which are involved in three functions such as endocrine secretion, sex hormone secretion and reproductive system disorder (Figure 7B and Supplementray Figure S12, S13). All the pathways were enriched in the comparison of 47XXY vs. 46XY (Figure 7B).

We next analyzed the pathway enriched genes using Sankey and Cytoscape (Figures 7C, D). We found that a large proportion (21/28, 75%) of them was CG upregulated genes (Figures 7C, D). Based on the KEGG analysis in combined WGBS and ATAC-seq, we found that the 47XXY individuals are recessive in hormone secretion and reproductive related pathway (Figures 7C, D).

Moreover, the thyroid hormone synthesis signaling pathway, GnRH signaling pathway, and estrogen signaling pathway displayed an abundance of DMR promoter genes in 47XXY individuals (Supplementray Figures S11, S14–S16 and Supplementray Tables S19, S20). We next constructed the network analysis of these four hormone secretion pathways. Compared to 46XX, *SRC* and *PRKX* were abundant in the thyroid hormone, GnRH, and estrogen pathways in 47XXY individuals. And *LHB*, an important gene in the GnRH signaling pathway, showed a hyper methylation level in 47XXY.

In summary, the extra Chr.X induces whole genome changes, and altered sexual hormone pathways in 47XXY.

## Discussion

The presence of an extra Chr. X in 47XXY subjects can cause altered levels of gene expression and abnormal phenotypes. We here described the genome-wide DNA methylation landscape from plasma sampels of 47XXY adults, and chromatin accessibility maps from 47XXY fetal amniotic cells. We found that the abnormal DNA methylation occurs throughout the whole genome, especially in the Chr. X in 47XXY individuals. The 47XXY amniotic cells show a dynamic higher chromatin accessibility than 46XX and 46XY. We also detected altered methylation and chromatin accessibility in *XIST*, XCI-enriched and XCIE region, and subtle differences in PAR and Y-linked region in 47XXY individuals. Moreover, the whole-genome methylation changes in 47XXY results in altered regulation of genes associated with sexual hormone secretion. We speculate that the abnormal opening of chromatin in the early stages may induce the whole genome methylation changes in 47XXY.

Recent studies of 47XXY have attempted to address transcriptomics in multiple cells (Winge et al., 2018a; Winge et al., 2018b; Mahyari et al., 2021). The presence of aberrant chromosome material may cause epigenomic instability by changing the regulation of transcription (Passerini et al., 2016). The dosage compensation of the extra Chr. X (47XXY) is associated with abnormal phenotypes such as taller than average height, low levels of fertility, azoospermia, and elevated gonadotropin levels (Hunt et al., 1998; Samango-Sprouse, 2001; Pessia et al., 2012; Sun et al., 2013; Belling et al., 2017; Sahakyan et al., 2017; Raznahan et al., 2018; Skuse et al., 2018). It is still unknown about how epigenetic regulatory landscapes evolve in 47XXY. The degree of methylation in the 47XXY group tends to be in between the amount of methylation detected in the 46XY and 46XX groups (hypomethylation to males and hypermethylation to females) (Viana et al., 2014; Sharma et al., 2015; Wan et al., 2015; Skakkebaek et al., 2018). In our study, we have found that the methylation level in 47XXY samples with the extra Chr. X is similar to that in 46XX samples. These results imply that an extra X in humans might cause epigenetic instability or alterations, which may be implicated in the phenotype seen in sex chromosome aneuploidies. In support of this hypothesis, compared to 46XY males, we found that the *O*-GlcNAc Transferase (OGT), located on the X-chromosome (Xq13) close to the X-inactivation center (XIC), is demethylated in 47XXY, suggesting that *OGT* levels may be controlled by dosage compensation (Chery and Larschan, 2014; Olivier-Van Stichelen et al., 2014; Olivier-Van Stichelen and Hanover, 2014). This strongly suggests that 47XXY individuals show similar CG levels to those in 46XX in Chr.X.

Previous studies have shown that aneuploidy leads to various genomic instability, such as pre-mitotic DNA errors, DNA damage, chromosome breaks, replication dynamics and genomic rearrangements (Joshua et al., 2011; Ariyoshi et al., 2016; Passerini et al., 2016; Li et al., 2017). The studies of 47XXY have mainly focused on gene regulatory and DNA methylation level (Viana et al., 2014; Sharma et al., 2015; Wan et al., 2015; Skakkebaek et al., 2018; Zhang et al., 2020). Whether the extra Chr.X causes abnormal chromatin accessibility in fetal stages is still unclear. Our results show that genes in *XIST*, XCI and XCIE-enriched region are abnormally active, which might be a main inducement of XXY phenotypes, and Y-linked genes are not significantly changed in the 47XXY group (Mroz et al., 1999; Park and Kuroda, 2001; Belling et al., 2017; Skuse et al., 2018). It is likely that some key genes in the Chr.Y such as *SRY* still play a stabilizing regulatory role to keep the first and secondary sexuality in 47XXY individuals (Arnold, 2012; Grinspon and Rey, 2016; Arnold, 2017).

Studies have shown that the *XIST* gene is more active in 47XXY than in 46XX individuals (Ji et al., 2015; Belling et al., 2017). Conversely, we here found that the XCI-enriched genes in 47XXY show a similar trend to that in 46XY in both CG and peak genes. In the XCIE, the peak genes in 47XXY are similar to those in 46XX, while the CG genes are similar to those in 46XY. Previous study suggested that lower Xp22.31 gene dosage in 47XXY males may increase their likelihood of exhibiting particular phenotypes relative to females (Davies, 2021). The *XIST* gene is more active in 47XXY than in 46XX individuals (Ji et al., 2015; Belling et al., 2017). Conversely, we found that the XCI-enriched genes in 47XXY show a similar trend to that in 46XY in both CG and peak genes. In the XCIE, the peak genes in 47XXY are similar to those in 46XX, while the CG genes are similar to those in 46XY. Previous study suggested that a lower Xp22.31 gene dosage in 47XXY males may increase their likelihood of exhibiting particular phenotypes relative to females (Davies, 2021). The unbalanced CG and peak calling may give an explanation for the increased prevalence in 47XXY of several conditions, which are usually more common in women (gynoid proportions and autoimmune diseases) (Zitzmann et al., 2004). Thus, the X dosage compensation may be associated with phenotypes in 47XXY. Further research will be needed to clarify the specific role of these elements in different tissues and cell types.

Generally, the 47XXY individuals have testicular failure with small testes and hyper gonadotrophic hypogonadism, and high serum levels of LH and FSH (Robinson et al., 1986; Wikstrom and Dunkel, 2008; Aksglaede and Juul, 2013; Davis et al., 2015; Davis et al., 2019). Testicular atrophy affecting primarily the tubular compartment and spermatogenesis. Markers that reflect the integrity of the blastic epithelium such as serum inhibin B, and anti-müllerian hormone (*AMH*) start to decrease from puberty and are undetectable in adults, whereas follicular stimulating hormone (FSH) tends to increase (Lanfranco et al., 2004; Aksglaede et al., 2011; Rohayem et al., 2015). Some new epigenetic markers from our results may be used for prediagnosis, such as *IGF1, ITPR2, MAD1L1, MAPK8* and *PLCB4*. Further study is required to investigate if these methylation changes also affect gene expression. Still, it is interesting to speculate that the abnormal hormone secretion may be possibly driven by epigenetic alterations.

In this study, we constructed the epigenetic landscape using two sequencing methods in 47XXY: WGBS and ATAC-seq. The methylation level and chromatin accessibility are altered in 47XXY individuals. The abnormal opening of chromatin in fetal cells may be related to the whole genome methylation changes and abnormal gonadotrophic hormone secretion seen in 47XXY. This work shows that the X dosages of gene regulation is deeply influenced by the coordination of epigenetic activities in whole genome regulatory architectures. Our study has provided a reference to find new epigenetic biomarkers for early diagnosis and therapy of Klinefelter Syndrome.

## Materials and methods

### Sample collection

The adult blood samples for the WGBS were obtained from Xiamen Maternal and Child Health Hospital. We used three 47, XXY blood samples, three 46, XX and three 46, XY blood samples with the similar ages (20-year-old) for the DNA extraction for the further WGBS analysis. Briefly, the samples with the following characteristics were excluded from this study, such as (1) poorly controlled blood pressure, hyperlipidemia and diabetes; (2) alcohol consumption and cigarette smoking; (3) infectious diseases.

The anonymized residual samples for ATAC-seq were obtained from Xiamen Maternal and Child Health Hospital. The amniotic fluid cells were collected from these samples of pregnant women undergoing genetic testing for routine clinical investigation. We selected two amniotic fluid cells with a karyotype identified as 47, XXY. The two 46, XY samples and two 46, XX samples were selected as the control groups. A nested case-control study was performed in amniocytes matched for fetal sex, maternal race/ethnicity, maternal age, gestational age at amniocentesis, and gestational age at birth.

This study was approved by the Institution Ethic Issue Committee of the First Hospital of Quanzhou (No. [2018]101).

### DNA quantification, qualification and library preparation

Genomic DNA degradation and contamination was monitored on agarose gels. DNA purity was checked using the NanoPhotometer^®^ spectrophotometer (IMPLEN, CA, United States). DNA concentration was measured using Qubit^®^ DNA Assay Kit in Qubit^®^ 2.0 Flurometer (Life Technologies, CA, and United States).

### Whole genome bisulfite sequencing (WGBS) and library construction

A total amount of 5.2 μg of genomic DNA spiked with 26 ng lambda DNA were fragmented by sonication to 200–300 bp with Covaris S220, followed by end repair and adenylation. Cytosine-methylated barcodes were ligated to sonicated DNA as per manufacturer’s instructions. Then these DNA fragments were treated twice with bisulfite using EZ DNA Methylation-GoldTM Kit (Zymo Research), before the resulting single-strand DNA fragments were PCR amplificated using KAPA HiFi HotStart Uracil + ReadyMix (2X).

Library concentration was quantified by Qubit^®^ 2.0 Flurometer (Life Technologies, CA, United States) and quantitative PCR, and the insert size was assayed on Agilent Bio analyzer 2100 system.

The library preparations were sequenced on an Illumina PE150 platform and 125 bp/150 bp paired-end reads were generated (Novogene Bioinformatics Technology Co., Ltd., Beijing, China).

### WGBS data processing and identification of differentially methylated CpGs

The remaining reads that passed all the filtering steps were counted as clean reads and all subsequent analyses were based on this. At last, we use FastQC to perform basic statistics on the quality of the clean data reads.

Bismark software (version 0.16.3) was used to perform alignments of bisulfite-treated reads to a reference genome (-X 700 --dovetail) (Krueger and Andrews, 2011). The sequencing depth and coverage were summarized using duplicated reads. The results of methylation extractor (bismark_methylation_extractor, no overlap) were transformed into bigWig format for visualization using IGV browser. The sodium bisulfite non-conversion rate was calculated as the percentage of cytosine sequenced at cytosine reference positions in the lambda genome. CGI (CpG island, which are on average 1000 base pairs (bp) long and show an elevated G + C base composition) (Cross et al., 1994), CGI_shore (CpG island shores are regions flanking CpG islands, upstream and/or downstream, by up to 2000 bp and with a lower GC content than islands), promoter, 5’-UTR, exon, intron, 3’-UTR and repeats region.

Differentially methylated regions (DMRs) were identified using the DSS software (Feng et al., 2014; Wu et al., 2015; Park and Wu, 2016). The core of DSS is a new dispersion shrinkage method for estimating the dispersion parameter from Gamma-Poisson or Beta-Binomial distributions. DSS possesses three characteristics to detect DMRs. According to the distribution of DMRs through the genome, we defined the genes related to DMRs as genes whose gene body region (from TSS to TES) or promoter region (upstream 2 kb from the TSS) have an overlap with the DMRs.

### Assay for transposase accessible chromatin with high-throughput sequencing (ATAC-seq) and library construction

ATAC-seq was performed as previously described elsewhere (Buenrostro et al., 2013; Buenrostro et al., 2015). Briefly, amniotic fluid cells were cultured and harvested to prepare a single-cell suspension. A number of cells (50,000 cells) were centrifuged for 5 min (at 500 × g, 4°C) and suspended in cold PBS. The supernatant was removed and discarded. The cell pellet was resuspended in cold lysis buffer followed by immediate centrifugation for 10 min at 500 × g, 4°C. The supernatant was removed and discarded and the nuclei pellet was resuspended in the transposition reaction mix for 30 min at 37°C. After this step, purification was done using a Qiagen MinElute PCR Purification Kit and then the transposed DNA was eluted in 10 µL elution buffer. PCR was performed to amplify the library for 15 cycles.

After amplification, these libraries were sequenced as PE150 sequencing (Vazyme Biotech Co., Ltd., Nanjing, China).

### ATAC-seq data processing and identification of differentially peak annotation

For each sample, ATAC-seq data, sequencing adapters and poor quality bases were trimmed from the sequencing reads by using Skewer (Jiang et al., 2014). Reads were mapped to the hg38 reference genome using Burrows-Wheeler Transform (BWA) (parameters -T 25 -k 18) (Li and Durbin, 2009). Coverage tracks were computed as fragments per million per base pair (FPM) using deepTools bamCoverage (parameters -bs 10 –normalized Using CPM–extend Reads–ignore Duplicates) (Ramirez et al., 2014). Peak-calling was performed by using Genrich (https://github.com/jsh58/Genrich) to analyze replicates.

We defined a consensus peak set for all samples by merging the overlapping peaks across different samples by using BEDTools merge (Quinlan and Hall, 2010). The consensus peaks count table was calculated for each sample by using feature Counts (parameters–F SAF–pignoreDup) (Liao et al., 2013). We used the count table for the consensus peak set as input data for DESeq2 and differential peaks were identified as those with log_2_^(FC)^ > 1 and *p*-value <0.05. The consensus peaks were assigned to nearby genes using ChIPseeker annotate Peak (Yu et al., 2015). We classified the annotated peaks into upstream 5 KB (up5K), exon, intron, downstream 5 KB (down5K) and intergenic regions on the basis of priority order. If a peak located in an exon of one gene, and an intron of another gene, this peak will be annotated in exon.

### Gene ontology and KEGG enrichment

Gene Ontology (GO) enrichment analysis of genes related to DMRs was implemented by the GOseq R package, in which gene length bias was corrected (Young et al., 2010). GO terms with corrected *p*-value less than 0.05 were considered significantly enriched by DMR CG and peak genes.

We used KOBAS software to test the statistical enrichment of DMR CG and peak genes in KEGG pathways (Mao et al., 2005).

GSEA is a software to calculate the enrichment score of gene sets *via* functional categories (http://software.broadinstitute.org/gsea/msigdb/index.jsp) (Subramanian et al., 2005; Joly et al., 2021). The GSEA considers experiments with genomewide expression profiles from samples belonging to two classes. Genes are ranked based on the correlation between their expression (log_2_^(FC)^) and the class (KEGG, GO, DO, Reactome) distinction by using any suitable metric (Subramanian et al., 2005).

The heatmap and Sanky analysis was performed using the OmicShare tools,a free online platform for data analysis (http://www.omicshare.com/tools).

## Data Availability

The datasets presented in this study can be found in online repositories. The names of the repository/repositories and accession number(s) can be found in the article/Supplementary Material. The released data presented in the study are deposited in the NCBI GEO datasets,accession number (GSE181854).
