# Supplementary material for: Integrative epigenome profiling of 47XXY provides insights into whole genomic DNA hypermethylation and active chromatin accessibility
Source: Front Mol Biosci. 2023 Mar 27;10:1128739. doi: 10.3389/fmolb.2023.1128739 (PMC10083376; doi:10.3389/fmolb.2023.1128739)
Supplement: Supplementary file 21 [file DataSheet1.DOCX]

**Supplementary for:**

**Integrative epigenome profiling of 47XXY provides insights into whole genomic DNA hypermethylation and active chromatin accessibility**

Nan Miao^1^, Zhiwei Zeng^1^, Trevor Lee^2^, Qiwei Guo^3^, Wenwei Zheng^4^, Wenjie Cai^5^, Wanhua Chen^6^, Jing Wang^1^ and Tao Sun^1,*^

^1^Center for Precision Medicine, School of Medicine and School of Biomedical Sciences, Huaqiao University, Xiamen, Fujian 361021 China.

^2^Department of Cell and Developmental Biology, Cornell University Weill Medical College, New York, NY 10065, USA.

^3^United Diagnostic and Research Center for Clinical Genetics, Women and Children’s Hospital, School of Medicine & School of Public Health, Xiamen University, Xiamen, Fujian, China.

^4^Quanzhou Women and Children’s Hospital, Quanzhou, Fujian China

^5^Department of Radiation Oncology, First Hospital of Quanzhou, Fujian Medical University, Quanzhou, Fujian China

^6^Department of Clinical Laboratory, First Hospital of Quanzhou, Fujian Medical University, Quanzhou, Fujian China

^*^**Corresponding author:** **Dr. Tao Sun**, Email: taosun@hqu.edu.cn.

**Tables:**

**Table S1** Quality control statistics of WGBS

**Table S2** mapping_summary of WGBS

**Table S3** genome_covg_summary of WGBS

**Table S4** Cytosines_covg_summary of WGBS

**Table S5** chromosomal density in CG level

**Table S6** 47XXY_vs_46XY_CG_mea46XYethy

**Table S7** 47XXY_vs_46XX_CG_mea46XYethy

**Table S8** Alig46XYent QC of ATAC

**Table S9** Pearson_atac

**Table S10** peak distribution

**Table S11** peak counts-47XXY_vs_46XY

**Table S12** peak counts-47XXY_vs_46XX

**Table S13** peak genes in sex chromosome of ATAC

**Table S14** CG_47XXY_vs_46XY _ATAC_methylation

**Table S15** CG_47XXY_vs_46XX_ATAC_methylation

**Table S16** ATAC_WGBS X&Y genes in four classes

**Table S17** 47XXY_vs_46XY.CG.DMR_genes.GO_enrichment_result

**Table S18** 47XXY_vs_46XX.CG.DMR_genes.GO_enrichment_result

**Table S19** KEGG analysis 47XXY_vs_46XY.CG.DMR_genes.identify

**Table S20** KEGG analysis 47XXY_vs_46XX.CG.DMR_genes.identify

**Figures:**

**Figure legend**

**Figure S1 PCA (A) and peason analysis (B) of WGBS**

**Figure S2 Circos graph of chromosomal methylation level**

A-C Circos graph of chromosomal methylation level in 46XY, 47XXY and 46XX groups.From the outside to the inside: linear methylation level, gene number density heat map, linear methylation density; internal rulers: three sequential enviro46XYents (CG in red, CHG in blue; CHH is purple), gene density thermal scale: gray to black indicates low to high number of genes.

**Figure S3 DMR genomic distributions in three sequential enviro46XYents (CG, CHG, CHH) of 47XXY vs 46XY(A) and 47XXY vs 46XX (B).**

**Figure S4 DMR length distributions in three sequential enviro46XYents (CG, CHG, CHH) of 47XXY vs 46XY(A) and 47XXY vs 46XX (B).**

The x-coordinate represents the LENGTH of DMR, the y-coordinate represents the density value at each length, and the black represents the distribution fitting curve.

**Figure S5 The violin map of horizontal distribution in CHH, CHG DMR methylation** **in 47XXY vs 46XY (A) and 47XXY vs 46XX (B)**

The horizontal coordinate represents the comparison group, and the vertical coordinate represents the methylation level value. The distribution of DMR methylation level is shown in the form of violin plot (inside is boxplot, and the flanking is the quantity distribution under this methylation level.

**Figure S6 Three sequential enviro46XYents (CG, CHG, CHH) of DMR in different functional regions of 47XXY vs 46XY (A) and 47XXY vs 46XX (B)**

The horizontal axis represents each functional region (CGI (CpG island), CGI_shore (CpG island shores are regions flanking CpG islands, upstream and/or downstream, by up to 2000 bp with a lower GC content than islands), promoter, UTR5, exon, intron, UTR3 and repeats region). The vertical axis represents the number of DMR in each region of hyper/ Hypo DMR.

**Figure S7 The heatmap of clustered CHG, CHH DMR methylation level in 47XXY vs 46XY (A) and 47XXY vs 46XX (B).**

The horizontal axis represents the comparison group, the vertical axis represents the methylation level value clustering effect, and the blue to red represents the methylation level from low to high.

**Figure S8 Circos map of CHG, CHH DMR methylation genes in 47XXY vs 46XY (A) and 47XXY vs 46XX (B).**

From outside to inside: (1) Hyper DMR statistic log5(|areaStat|); The higher the outward dot is, the more significant the position difference is. The red circle represents. (2) TE, and the heat map of the proportion of repeat original. (3) Gene density heat map. (4) Hypo DMR statistic log5(|areaStat|).

**Figure S9 PCA (A) and peason analysis (B) of ATAC-seq.**

**Figure S10 Genedepth of mapped pea47XXY in 46XY(A), 47XXY (B) and 46XX (C) .**

**Figure S11 The enriched GO items of DMR CG genes in 47XXY vs 46XY (A) and 47XXY vs 46XX (B)**

**Figure S12 The enriched KEGG items of DMR CG genes in 47XXY vs 46XY (A) and 47XXY vs 46XX (B)**

The bubble chart of KEGG analysis. Y-axis represents the pathway name，X-axis represents the Rich factor, the size of bubble represents the number of genes, and the color of bubble represents the Q-value.

**Figure S13 The enriched KEGG items of** **DMR promoter CG genes in 47XXY vs 46XY (A) and 47XXY vs 46XX (B)**

The bubble chart of KEGG analysis. Y-axis represents the pathway name，X-axis represents the Rich factor, the size of bubble represents the number of genes, and the color of bubble represents the Q-value.

**Figure S14 Functional identification of DMR promoter CG genes.**

(A) The bubble chart of KEGG analysis in 47XXY vs 46XY.

(B) CG genes in hormone secretion.

(C) Key genes network in hormone secretion.

(D) Key genes network in WNT.

**Figure S15 The** **hormone secretion pathway of DMR promoter gene.**

**(A)** KEGG analysis of DMR promoter genes in (47XXY vs 46XY).

**(B)** Network of hormone secretion-related genes.

**(C)** Network of the genes (47XXY vs 46XY, 47XXY vs 46XX) in four different hormone secretion pathways (thyroid hormone, GnRH, Estrogen and Serotonergic synapse signaling pathway).

**Figure S16 KEGG network of 47XXY vs 46XY CG genes in Estrogen signaling pathway.**

**Figure S1**

**
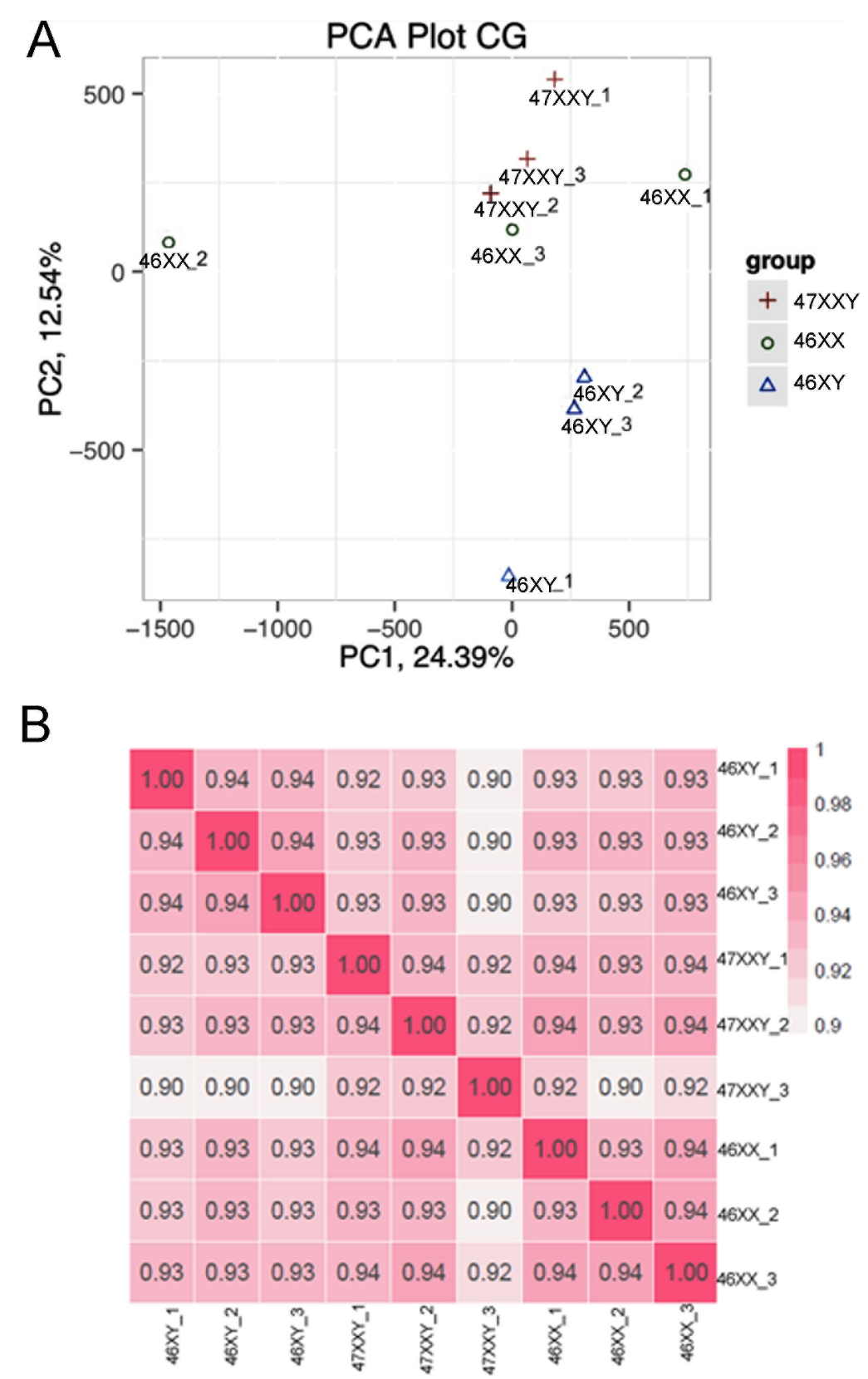
**

**Figure S2**

**
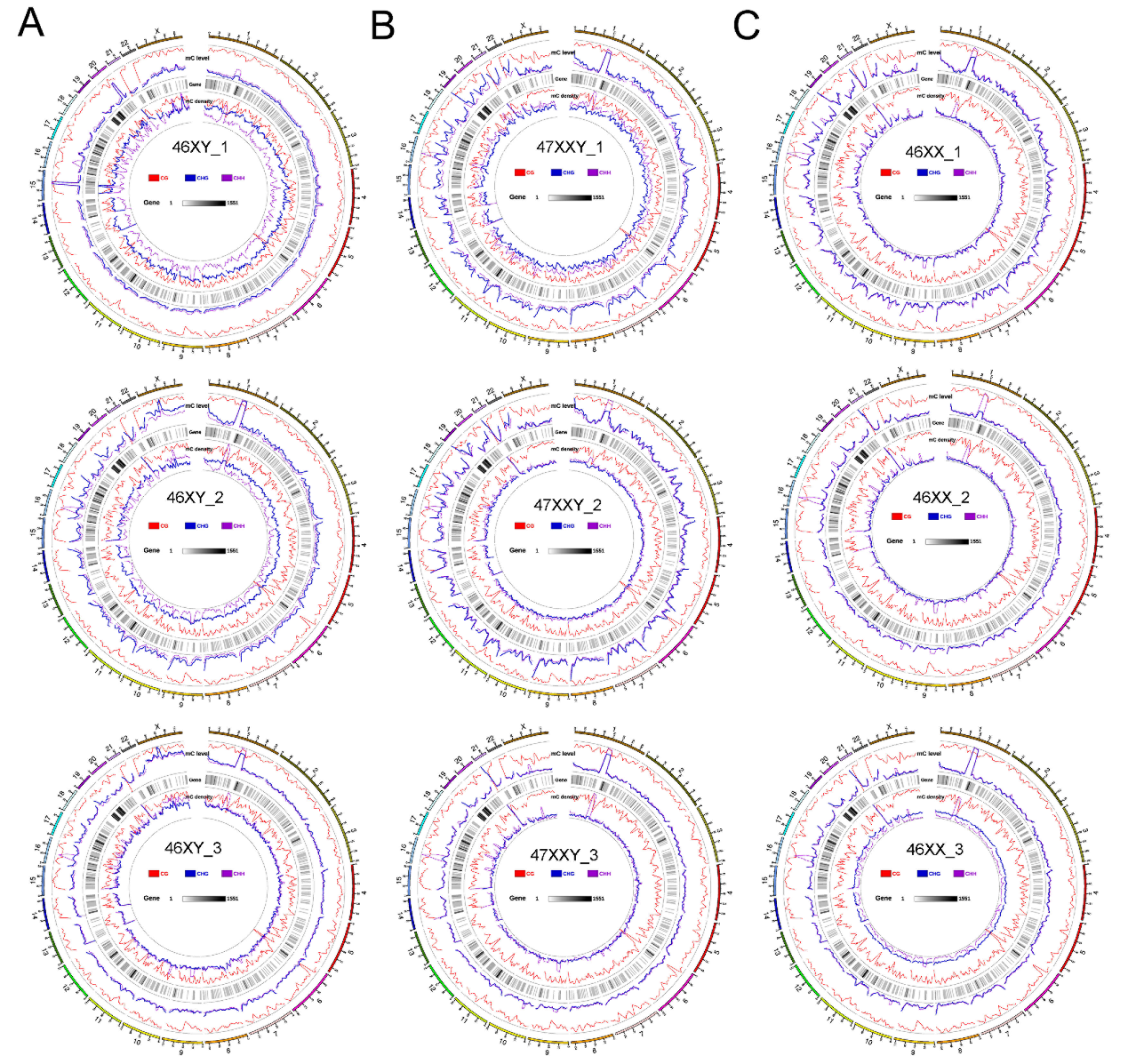
**

**Figure S3**

**
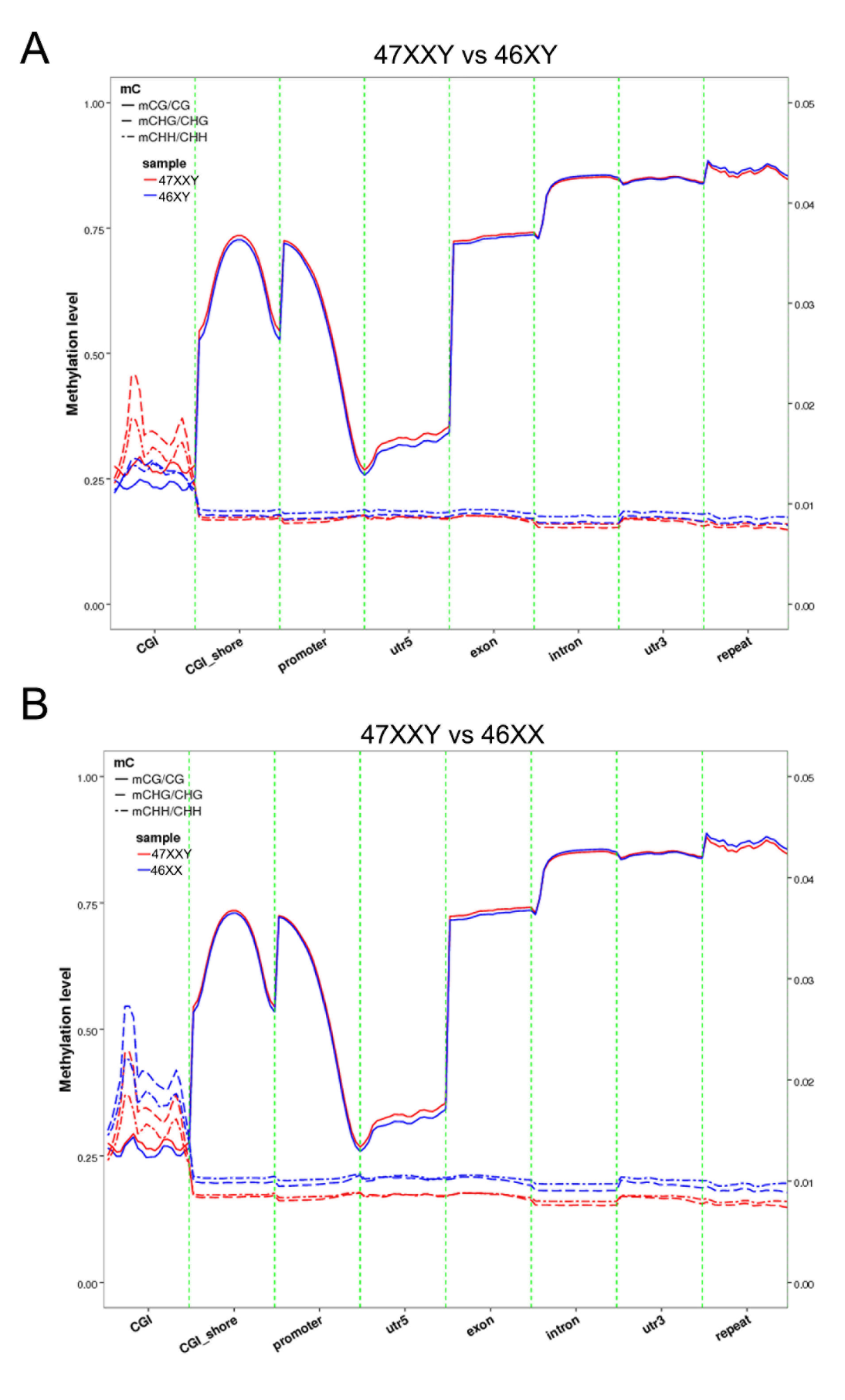
**

**Figure S4**

**
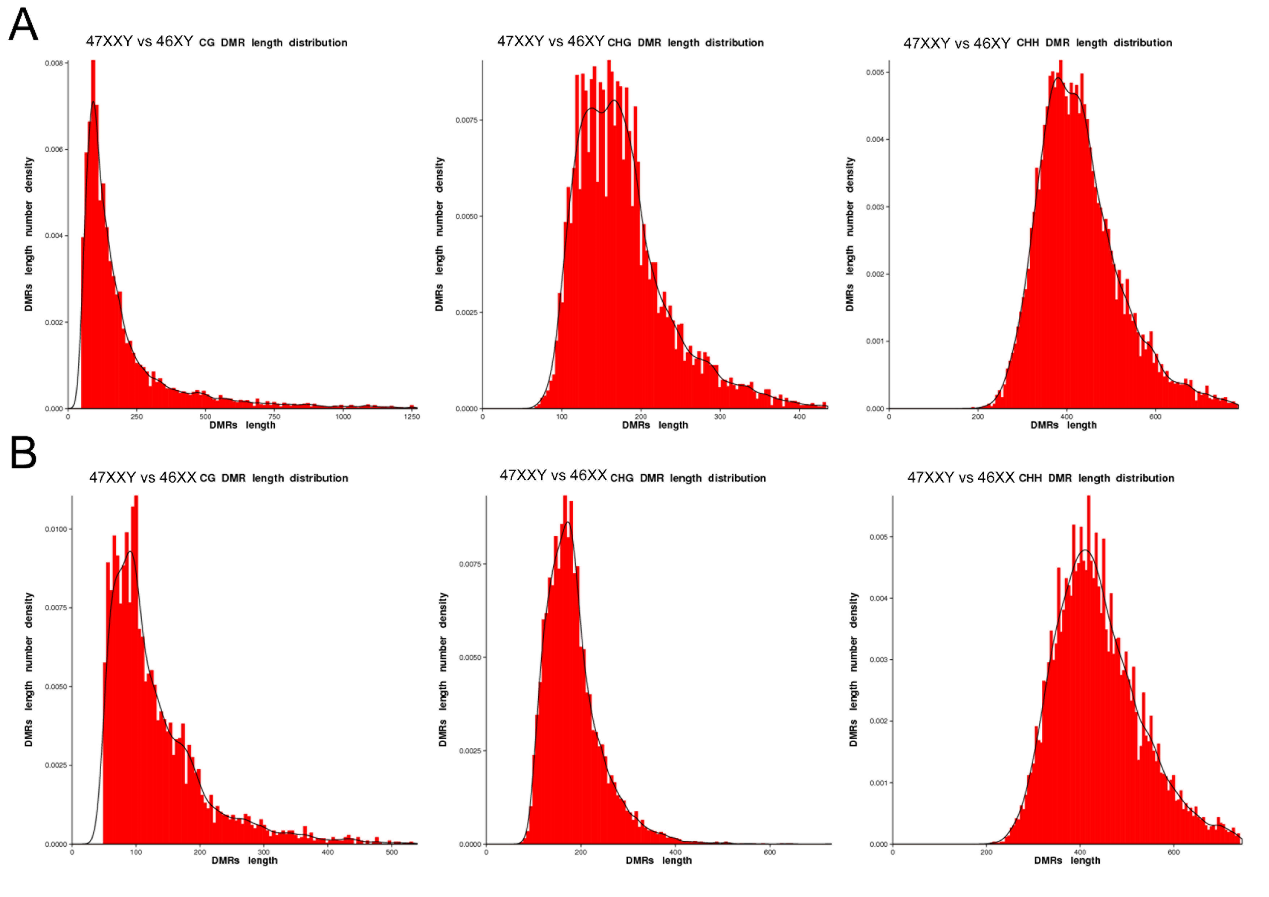
**

**Figure S5**

**
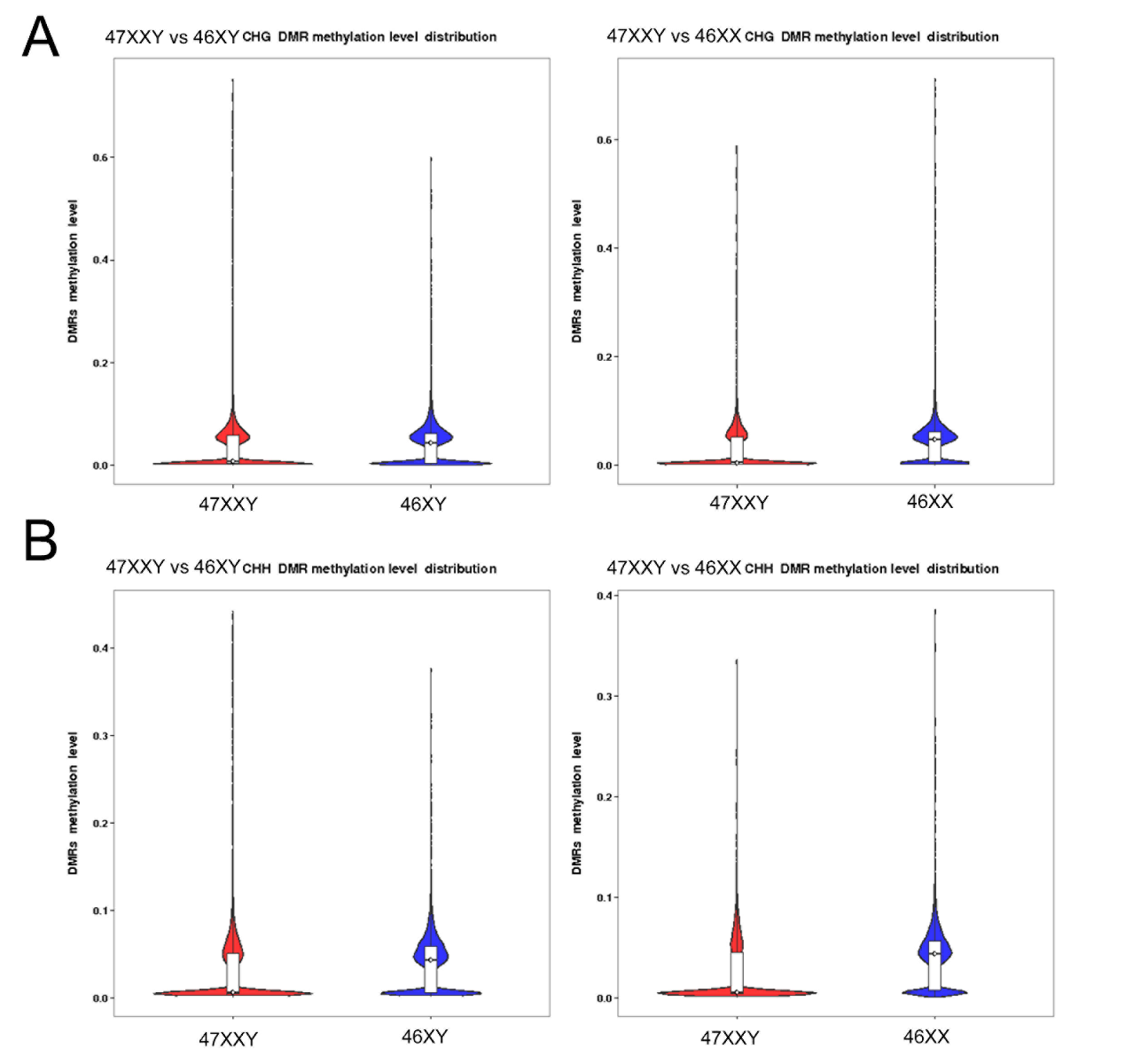
**

**Figure S6**

**
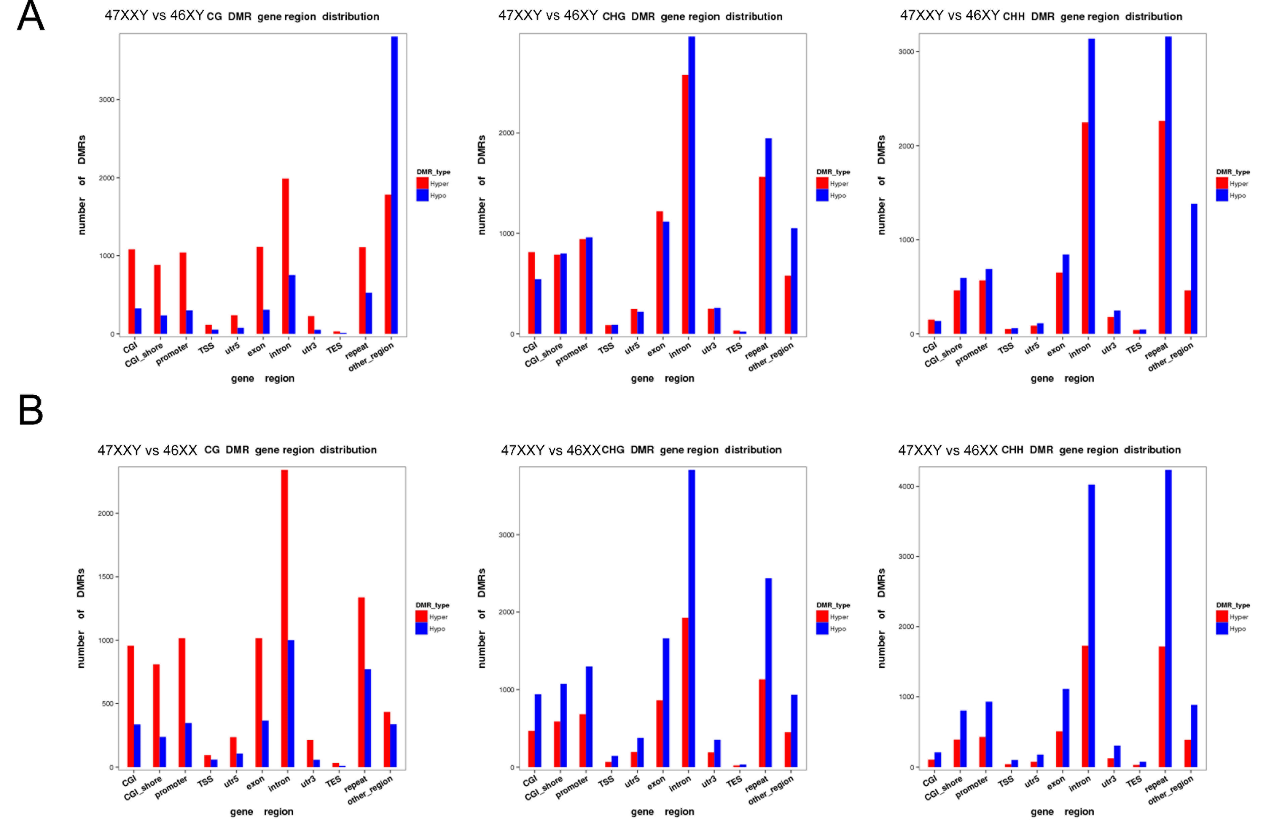
**

**Figure S7**

**
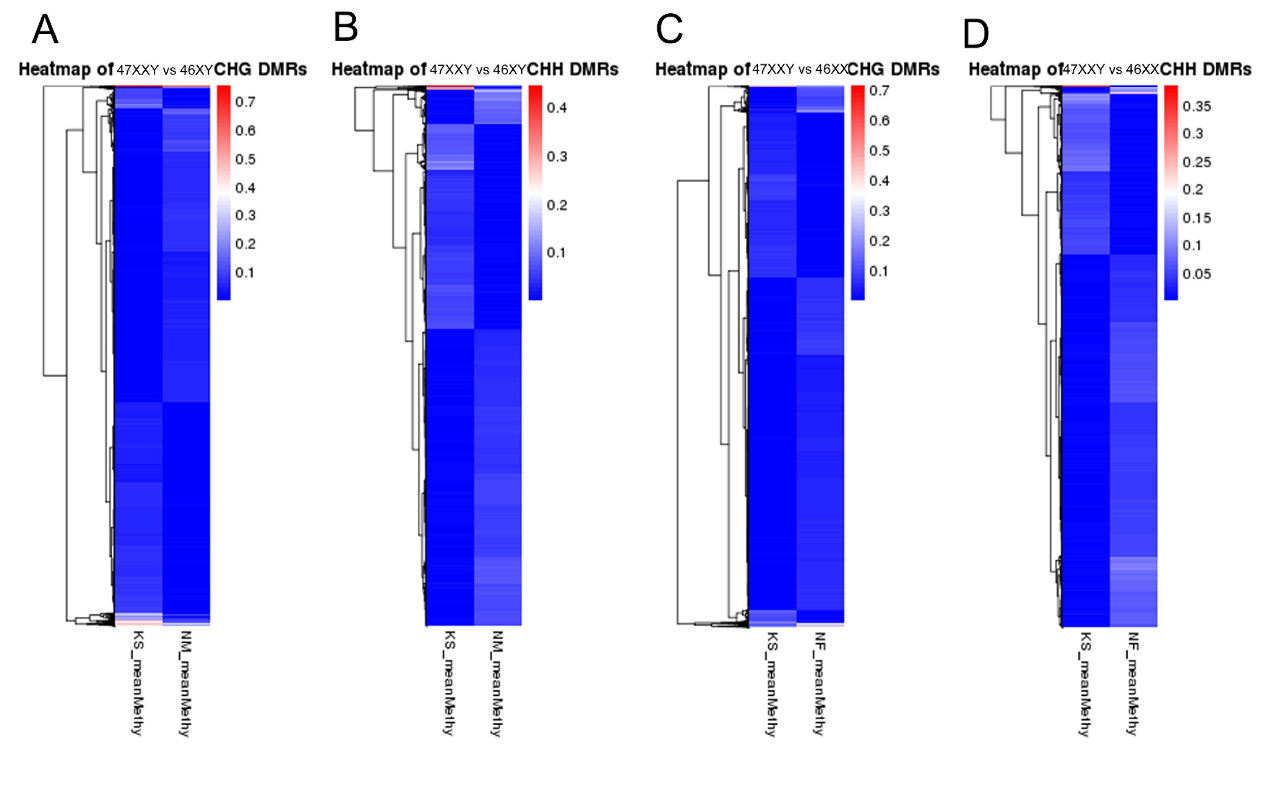
**

**Figure S8**

**
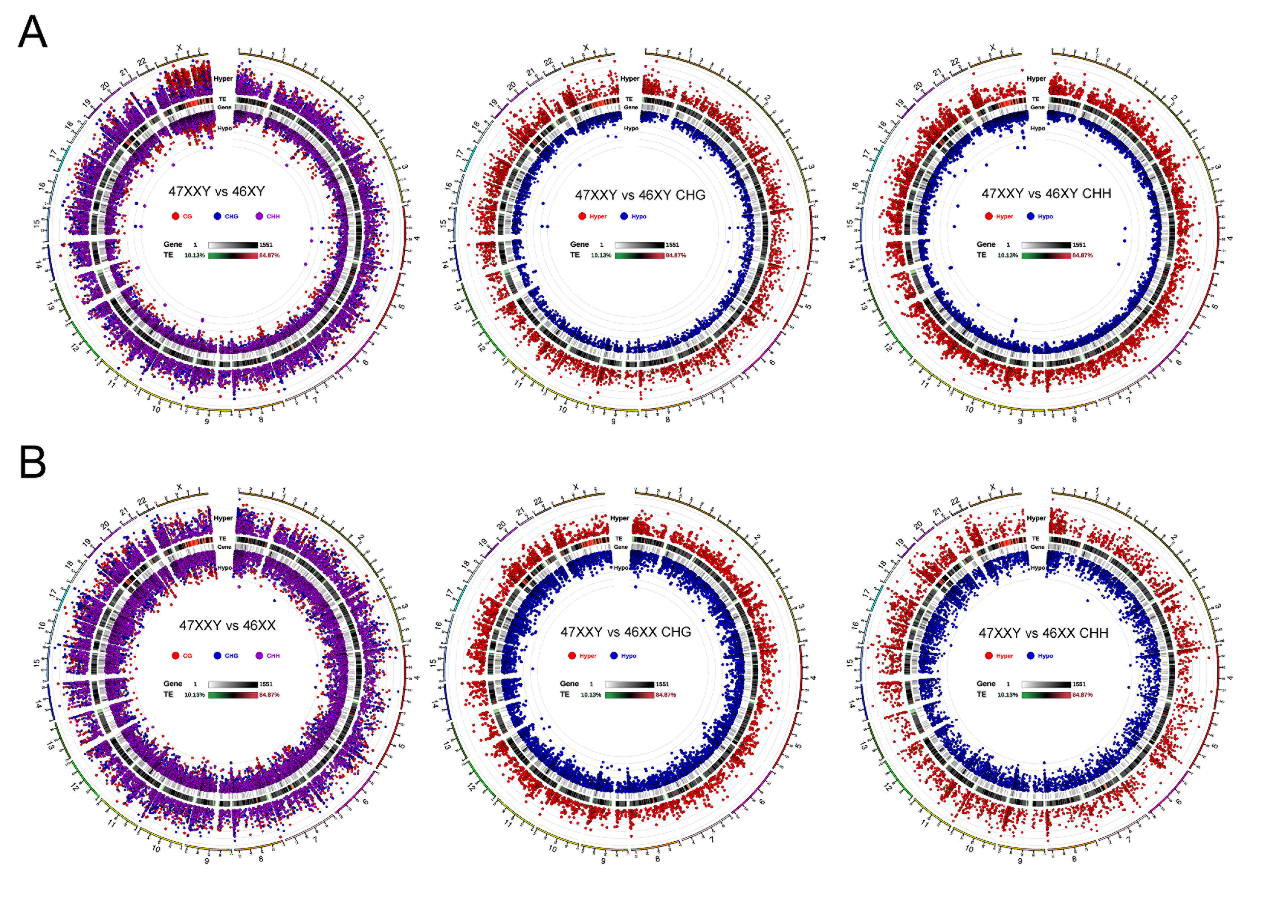
**

**Figure S9**

**
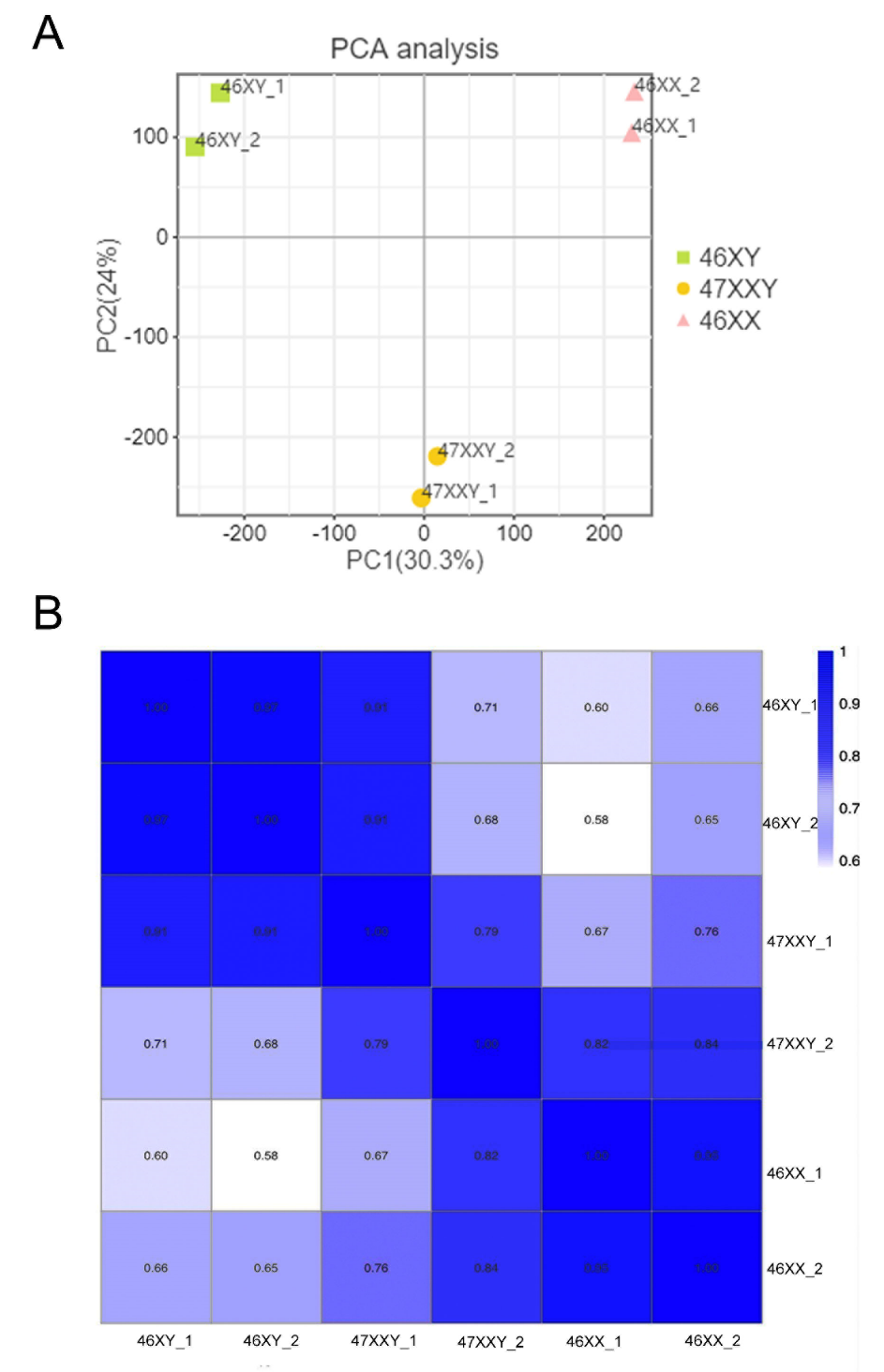
**

**Figure S10**

**
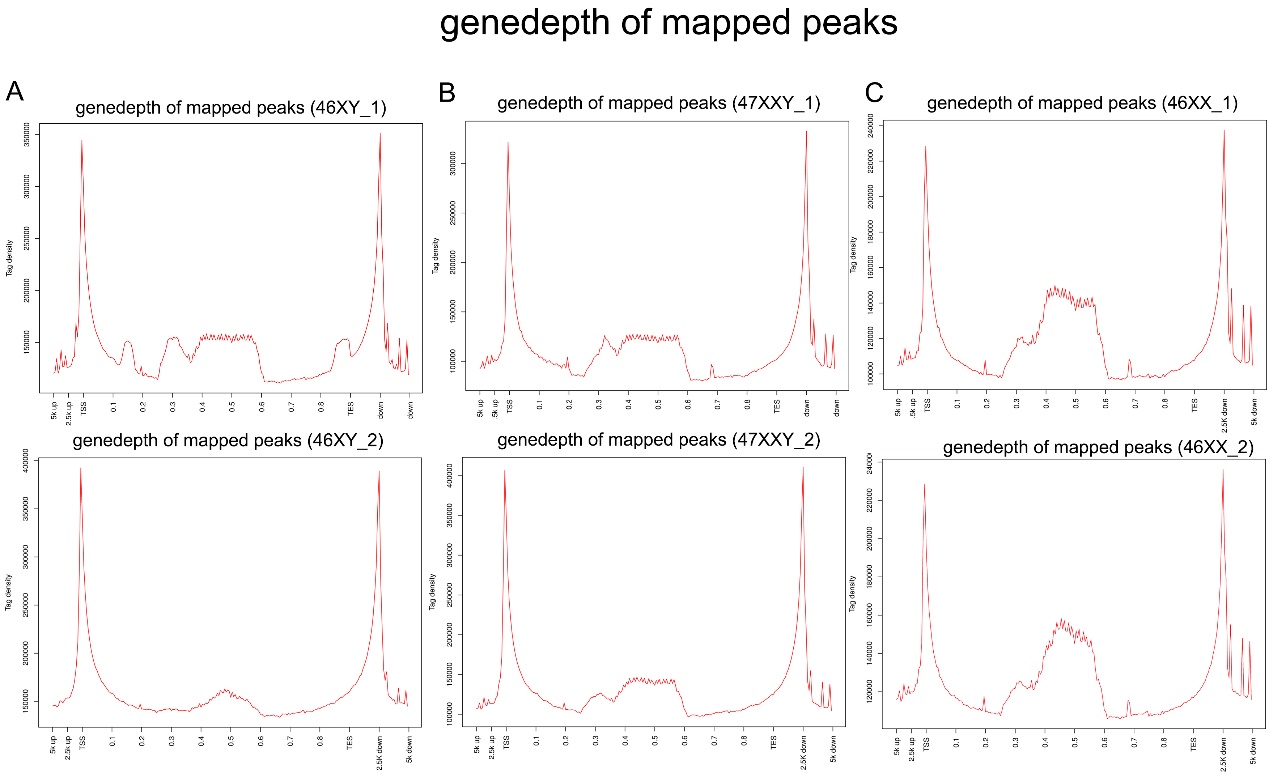
**

**Figure S11**

**
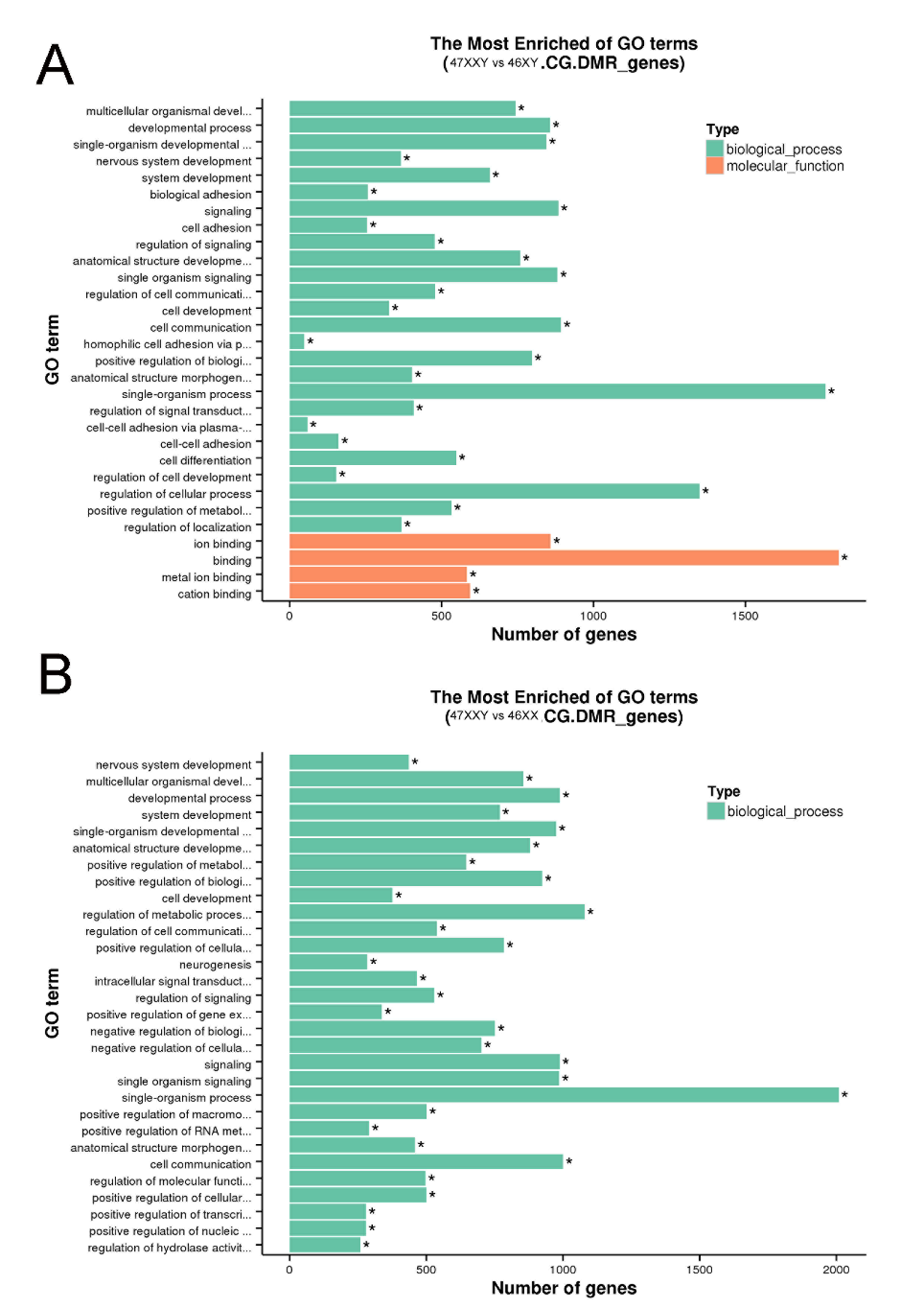
**

**Figure S12**

**
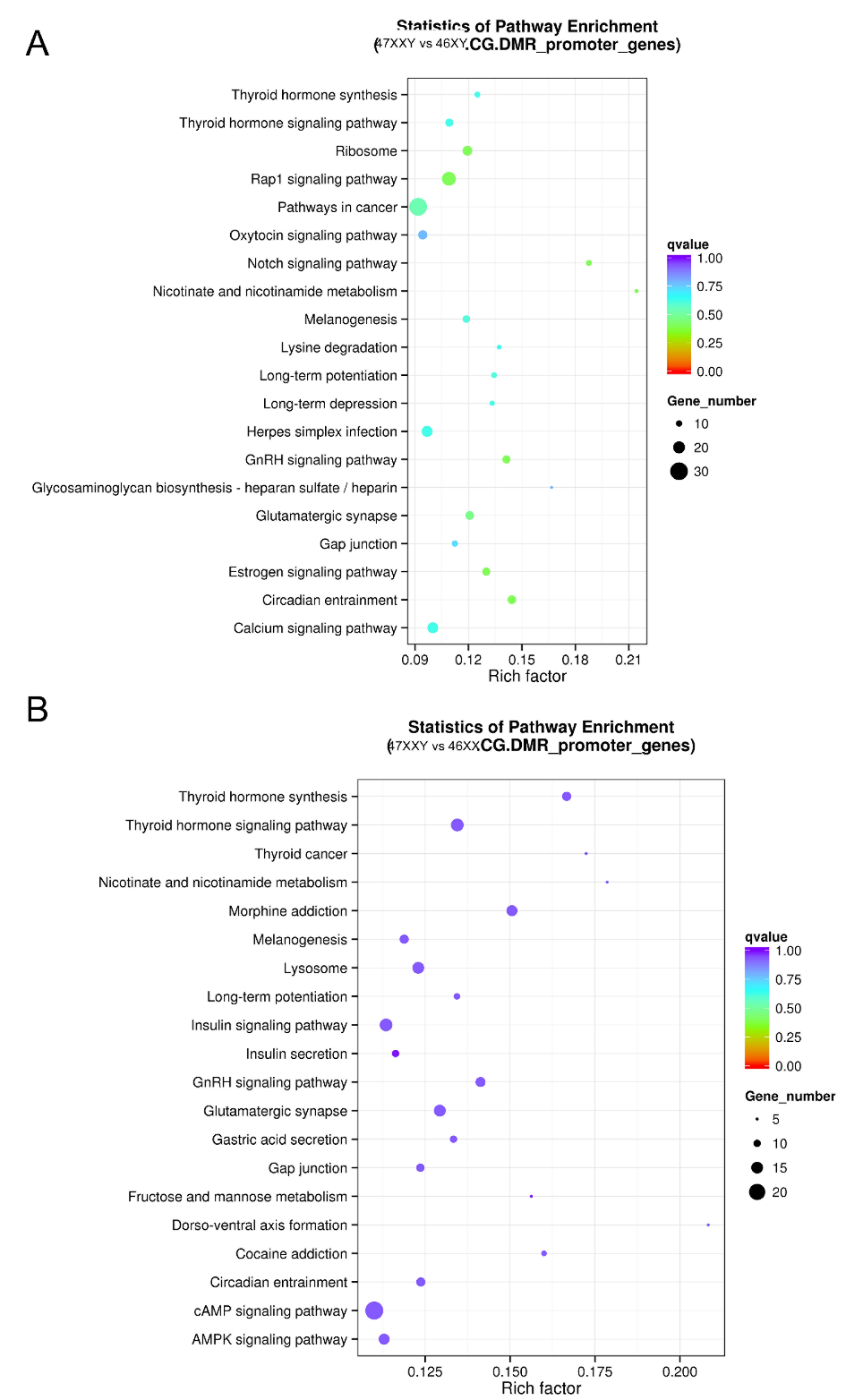
**

**Figure S13**

**
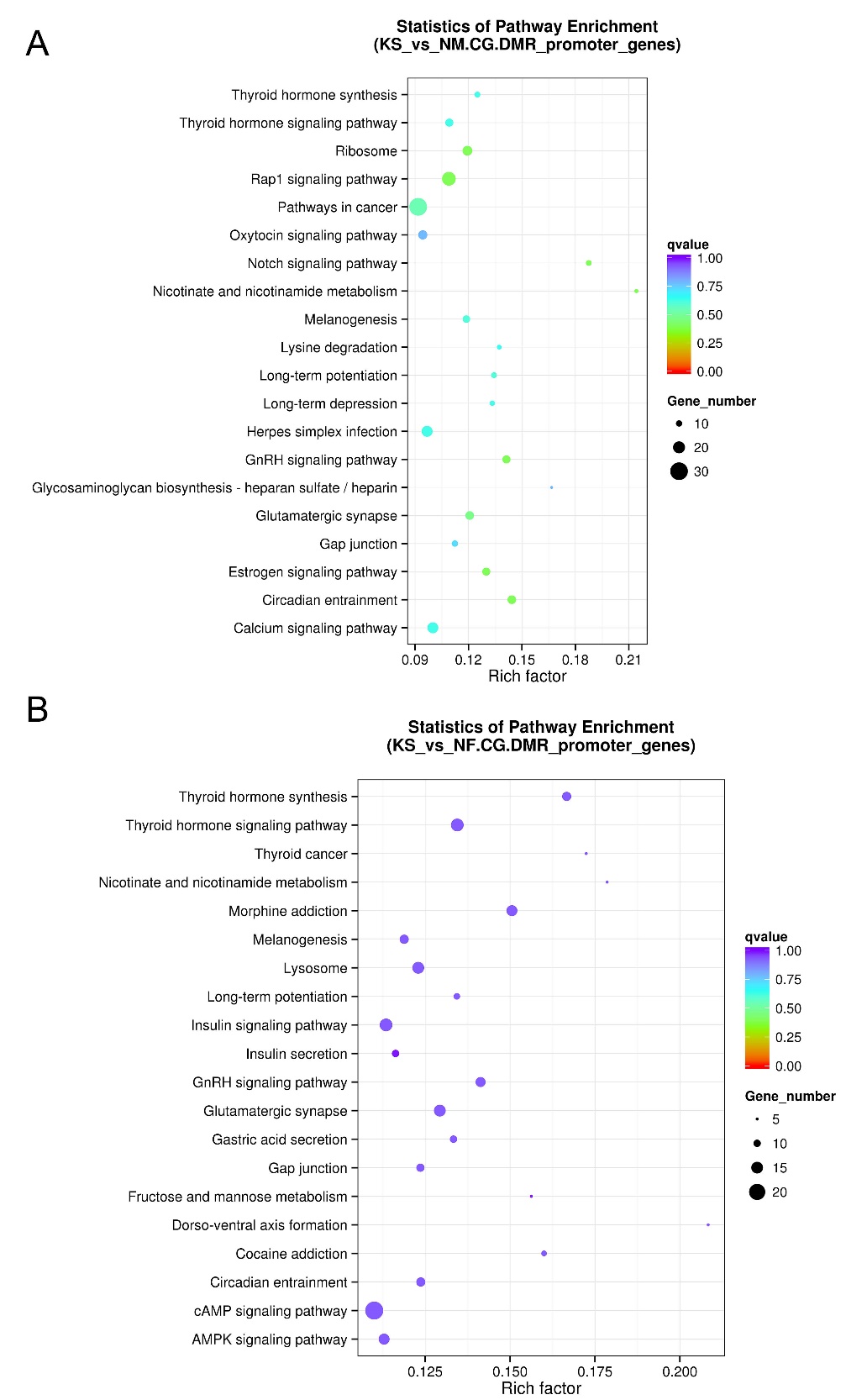
**

**Figure S14**

**
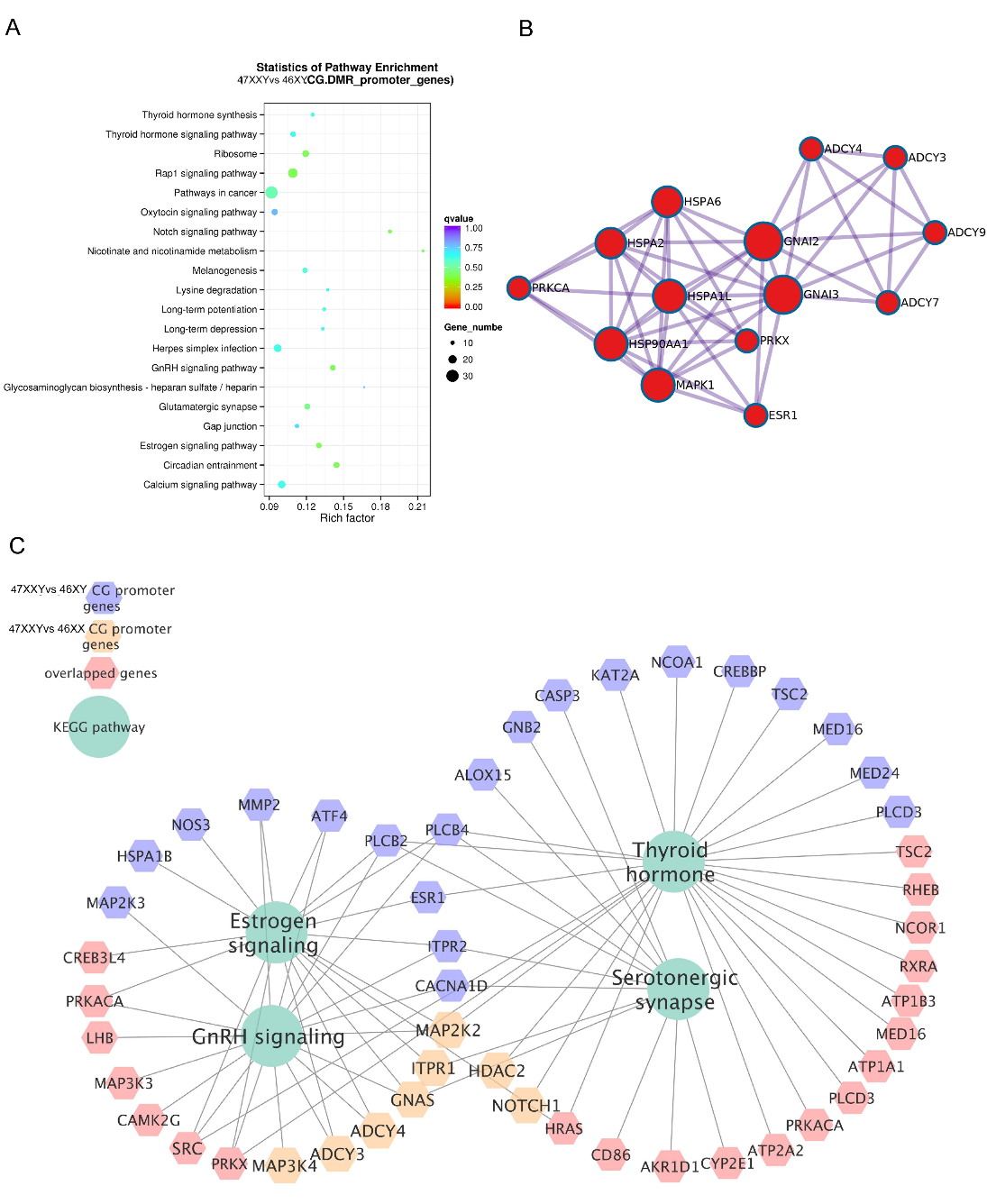
**

**Figure S15**

**
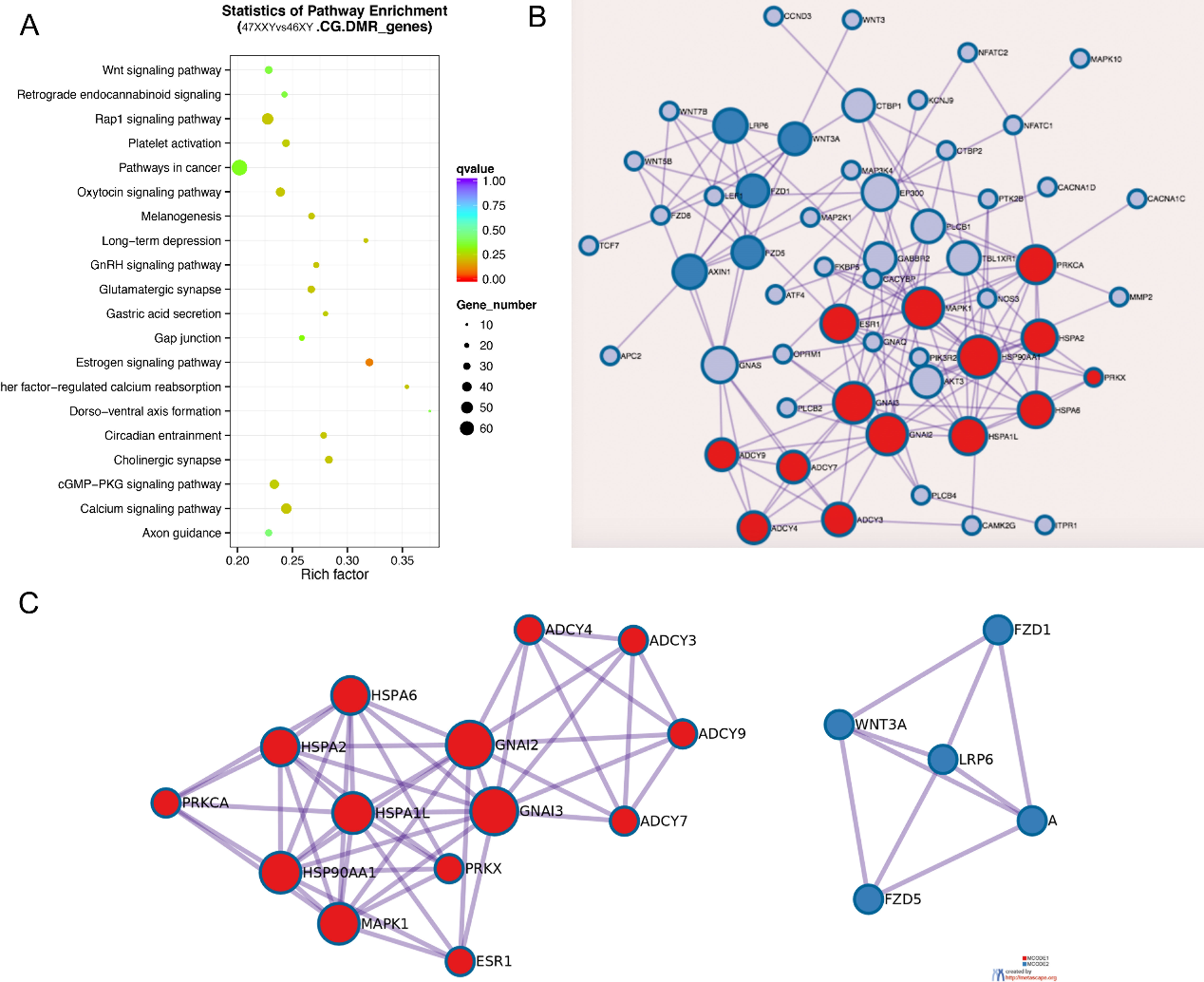
**

**Figure S16**

**
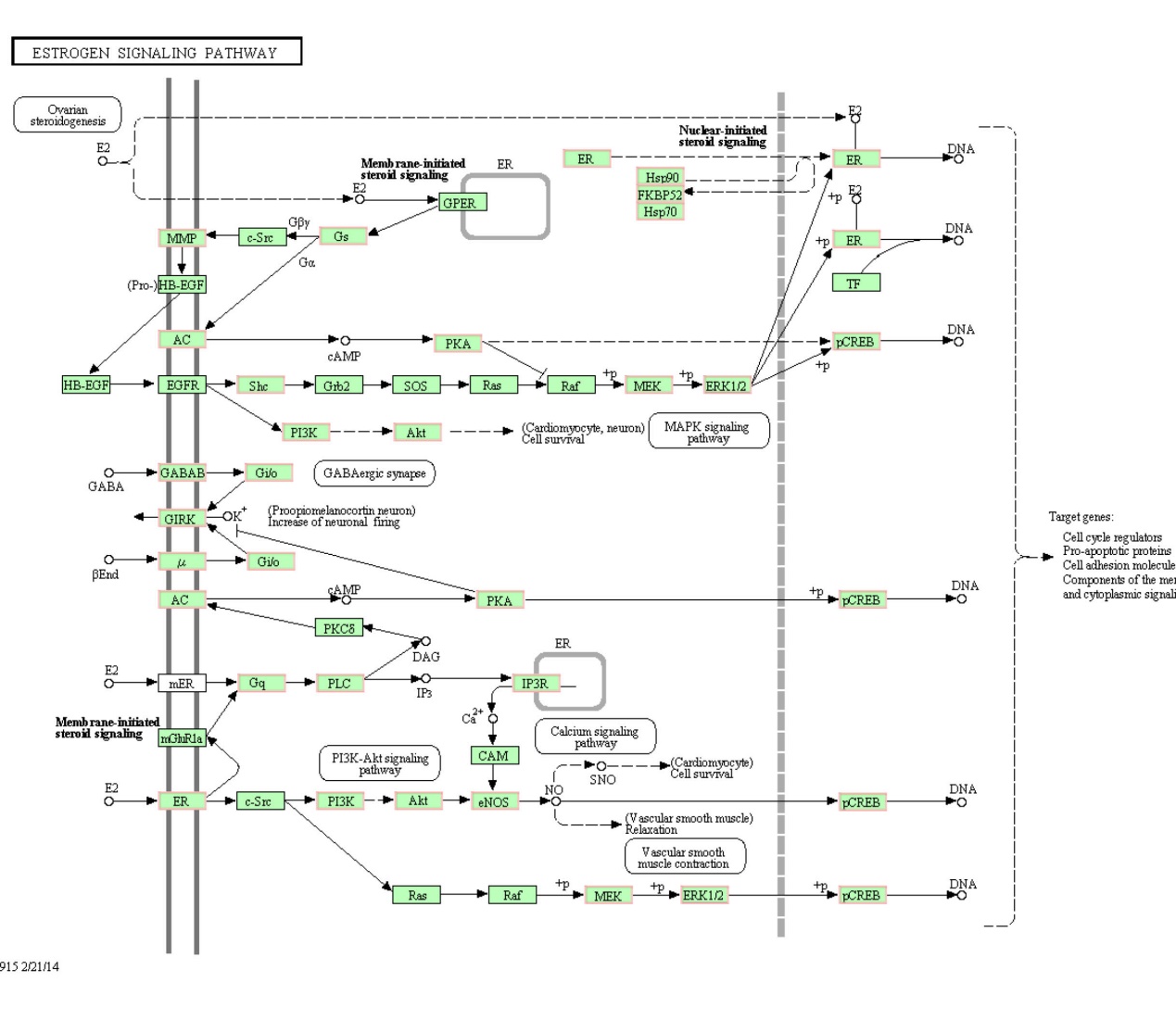
**
